# Reduced graphene oxide/ bismuth tungstate-based photocatalysts for enhanced dye photodegradation and photoelectrochemical water splitting†

**DOI:** 10.1039/d5ra04049c

**Published:** 2025-07-25

**Authors:** Amr Awad Ibrahim, Doaa A. Kospa, Salah Orabi, Salma M. Abo Kamar, Ahmed A. Salah, E. A. El-Sharkawy, S. A. El-Hakam, Awad I. Ahmed

**Affiliations:** a Department of Chemistry, Faculty of Science, Mansoura University Al-Mansoura Egypt amr_awad@mans.edu.eg; b Department of Chemistry, Faculty of Science, Suez University Suez Egypt

## Abstract

Semiconductor-based heterogeneous photocatalysis generates highly reactive charge carriers under solar illumination, making it a successful approach for wastewater purification and energy production applications. Herein, a simple solvothermal approach was used to generate a promising nanocomposite photocatalyst, reduced graphene oxide/bismuth tungstate (rGO/Bi_2_WO_6_). Various analytical tools were utilized to characterize the as-prepared catalysts. The oxidation of methylene blue (MB) and rhodamine B (RhB) dyes in the presence of solar light was performed to assess the photocatalytic activity of the rGO/Bi_2_WO_6_ combination. Also, photoelectrochemical (PEC) water splitting as an efficient and cost-effective way of producing hydrogen from water using solar energy is tested. The effect of the calcination temperature of Bi_2_WO_6_ and the amounts of graphene oxide on the catalytic activity was studied. After 30 minutes of exposure, the synthesised 10rGBW calcined at 700 °C (10rGBW-IV) showed good photodegradation percentages of 100.0% and 87.6% for MB and RhB dyes, respectively. Also, the larger photocurrent response intensity and lower arc radius of the electrochemical impedance of rGO/Bi_2_WO_6_ compared to Bi_2_WO_6_ revealed the synergistic effect on Bi_2_WO_6_ visible light responsiveness. According to the Mott–Schottky curve, 10rGBW-IV demonstrates a larger negative shift of the Fermi level (FB = −0.08 V *versus* RHE), indicating a stronger oxidation potential for water splitting.

## Abstract 2

Semiconductor-based heterogeneous photocatalysis generates highly reactive charge carriers under solar illumination, making it a successful approach for wastewater purification and energy production applications.

## Graphical abstract

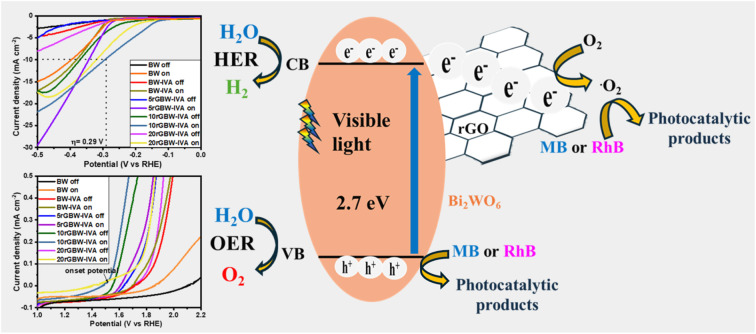

## 1 Introduction

The current emphasis on sustainable, renewable energy and clean water has increased due to the freshwater and energy crisis. Given the rising need for water to sustain socioeconomic progress and human health, water pollution is a major problem for researchers. As a result, water pollution and changes in the climate constitute major contributors to water shortages.^1^ Various dyes are utilized for many reasons in a variety of sectors, including cosmetics, concrete, plastic, rubber, textiles, food, printing, medicine, and the paper industry.^2,3^ These enterprises produce a large volume of effluent that contains carcinogenic and poisonous dyes, contaminating the water and making it unsuitable for human usage.^4^ Dyes considerably damage water quality due to their toxicity and non-biodegradability, posing serious hazards to aquatic ecosystems and environmental health.^5^ According to the United Nations Organization (UNO), contamination of drinking water by microorganisms and chemicals threatens the health of nearly half the population in developing countries. Numerous traditional methods are proven for dye removal from water, like electrochemical treatment,^6^ coagulation,^7^ membrane filtration,^8^ adsorption,^9^ and biodegradation.^10^ However, the broad implementation of these advanced water treatment systems is restricted by high operational costs, limited treatment capacity, and excessive reliance on chemical inputs. Renewable energy has recently become cost-competitive with fossil fuels, potentially saving trillions of dollars, lowering greenhouse gas emissions and improving energy security.^11^

Since it can remove pollutants even after continuous irradiation, the advanced oxidation process (AOP) utilizing heterogeneous photocatalysis is an effective method for removing pollutants.^12^ According to several researchers, photocatalysis is a viable and sustainable technology for wastewater treatment because it uses visible light, an available and environmentally safe energy source, to drive the reaction. The other wastewater treatment methods produce secondary pollution; hence, the photocatalytic technique of organic pollutant removal is taken into consideration owing to its cost-effectiveness and non-hazardous properties.^13–15^ Photocatalysis is a chemical reaction driven by absorbed light energy in the presence of a catalyst, producing electron–hole (e^−^/h^+^) pairs and free radicals which have a high ability to degrade many types of organic contaminants.^16,17^ Visible light photocatalyst can use a wider spectrum of electromagnetic radiation than ultraviolet (UV) light, which is more energy-efficient and economical. In visible light-driven photocatalysis, charge carriers interact with water and oxygen to produce reactive species, including superoxide (˙O_2_^−^) and hydroxyl (˙OH) radicals.^18^ These radicals help to break down organic pollutants in water.

Furthermore, the demand for renewable and sustainable energy sources has risen to limit the environmental pollution caused by CO_2_ emissions from the combustion of fossil fuels.^11^ Because of its high energy density, hydrogen is a clean and sustainable energy source that can meet the world's energy needs. Photoelectrochemical (PEC) water splitting is an efficient approach for cost-effective hydrogen production from water under solar irradiation.^19^ The PEC efficiency can be enhanced through several methods, including the improvement of light absorption and the reduction of catalyst recombination. Improving light absorption and reducing catalyst recombination are two strategies to boost PEC water-splitting efficiency. Significant work has gone into adjusting the valence band (VB) and conduction band (CB) edge locations of various compound semiconductors (also known as “bandgap engineering”) to adjust their interfacial energetics to certain photo-oxidation or photo-reduction processes, respectively.^20,21^ Several efforts have been made to exploit novel and diverse light sources and nanomaterials for organic waste removal by photodegradation. Doping and compositing are two methods for improving the structural characteristics and photodegradation performance of produced nanomaterials.^22,23^ The energy bandgap of these materials may be changed by shifting the band edge locations, which may then improve photocatalytic activity. A similar change can lead to bandgap narrowing, boosting solar light absorption while maintaining the redox potential of photogenerated charge carriers.

Metal tungstates have gained high attention among the numerous types of semiconductor photocatalysts.^24^ Metal tungstates are an intriguing class of inorganic compounds that exhibit the typical scheelite (MWO_4_) and wolframite (MWO_6_) formula that may be distinguished by the letter M.^25,26^ Pb, Sr, Ba, and Ca are examples of bivalent cations with large radii that have scheelite-type tetragonal structure, whereas M = Zn, Cu, Co, Mn, and Cd are examples of bivalent cations with smaller radii that have wolframite type monoclinic structure.^27,28^ For the scheelite-type structure, each tungstate group is bound by four oxygen atoms, but in the case of the wolframite-type structure, each tungstate atom is bound by six oxygen atoms.^29^ Bi-based photocatalysts have gotten a lot of attention lately.^24,30^ Among Bi-based semiconductors, Bi_2_WO_6_ is said to be a potent photocatalyst for air and wastewater purification. Because the octahedron of ceratoid WO_6_ is positioned in the sandwich of (Bi_2_O_2_)^2+^ and may increase the separation of photo-generated charges, Bi_2_WO_6_ is also stable with high activity.^31–33^ However, because of its poor light absorption, quick recombination of photo-generated e^−^/h^+^ pairs, and difficult migration, pure Bi_2_WO_6_ photocatalytic activity is constrained.^34^ To boost the photoinduced electron–hole pair separation of Bi_2_WO_6_, numerous effective techniques and technologies have been described, including doping,^35^ substitution,^36^ heterostructure creation with a narrow-band gap semiconductor,^37^ and coupling with a carrier.^38,39^ The bandgap of Bi_2_WO_6_, an n-type semiconductor with a structure made up of perovskite layers and a member of the Aurivillius family, is around 2.75 eV.^40–42^ Due to the wide band gap of Bi_2_WO_6_, it has low light absorption and thus limits catalytic activity. Several methods can be used to enhance the Bi_2_WO_6_ band gap, including defect engineering, deposition of metals and non-metals, and coupling or ion doping with different semiconductors.^43,44^

Compared to pure oxide nanostructure, semiconductor-based composites demonstrate improved photocatalytic activity.^45,46^ Additionally, graphene has gained a lot of interest for supporting catalytic nanoparticles due to its great thermal stability, mechanical strength, large specific surface area, superb electronic conductivity and excellent adsorption capacity.^47,48^ The addition of graphene to composites may provide ordinary properties that open up new possibilities for designing and creating future catalysts.^49^ In recent years, the production of metal nanoparticles on graphene *via* the reduction of metal precursors in the presence of suspensions of pure or exfoliated graphene has been shown. Additionally, a lot of composite materials were created using graphene, and the resulting composites showed a variety of benefits and were used in a variety of industries. Examples of modern composites that have been made and used in many fields are TiO_2_/MCM-41,^50^ polyaniline/reduced graphene oxide,^51^ molybdophosphoric acid/MCM-41,^52^ ZnO doped reduced graphene (rGO-ZnO),^53^ FeVO_4_/rGO/FeVO_4_,^54^ CuS/g-C_3_N_4_/rGO,^55^ and others. These materials have been synthesized using various methods and used as effective photocatalysts. As rGO serves as an electron acceptor and avoids electron–hole recombination, its addition to semiconductors is anticipated to decrease charge carrier recombination and boost photocatalytic efficiency.^56,57^

In this study, rGO-B_2_WO_6_ samples were generated by mixing B_2_WO_6_ calcined at different temperatures under the hydrothermal method. The hydrothermal method was effectively employed to reduce GO to rGO. Also, various samples with rGO content on B_2_WO_6_ were prepared. The synthesized nanocomposites are projected to produce several photo-generated e^−^/h^+^ pairs, hence increasing the photocatalytic efficiency of rGO-B_2_WO_6_ hybrids. Several factors are examined in our research to determine the efficiency of photodegradation performance involving concentration of the photocatalysts and organic pollutants, pH of the solution, reaction time and calcination temperature. Moreover, the materials were used as photocatalysts for the photoelectrochemical water splitting. We anticipate that our study will motivate additional research into the development of new photocatalysts for the efficient removal of catalyst residues and other hazardous pollutants from polluted water.

## 2 Experimental

### 2.1. Materials

Sulfuric acid H_2_SO_4_ (98%), sodium nitrate (NaNO_3_), hydrogen peroxide (H_2_O_2_, 30%), hydrochloric acid (HCl), sodium tungstate 2-hydrate (Na_2_WO_4_·2H_2_O), zinc nitrate hexahydrate (Zn(NO_3_)_2_·6H_2_O), isopropoanol, rhodamine-B (RhB), graphite (99.95% purity), sodium sulphate (Na_2_SO_4_), benzoquinone, ethylenediaminetetraacetic acid disodium salt, potassium hydroxide (KOH), ethanol (C_2_H_5_OH), Nafion (5 wt%), and methylene blue (MB) were all obtained from Sigma-Aldrich. Without any additional processing, all reagents were of analytical quality.

### 2.2. Photocatalysts preparation

#### 2.2.1. Graphene oxide (GO) preparation

Following the modified Hummer technique,^48,58^ 50 mL of H_2_SO_4_ (98%) was added to the mixture of graphite powder (1.0 g) and NaNO_3_ (1.0 g) and kept under vigorous stirring for two hrs in an ice bath. Subsequently, KMnO_4_ (5.0 g) was added to the mixture and stirred in an ice bath (below 10 °C) for 4 h. After that, the mixture was removed from the ice bath and then swirled at room temperature until the formation of brownish paste. After stirring for 24 h, hot DI water (350 mL) was then added slowly, followed by the addition of 650 mL of cold DI water. The colour of the solution turned brown when the reaction temperature was quickly raised and maintained below 98 °C. The process was eventually stopped by the H_2_O_2_ addition (30 mL), which caused the solution to become yellow, and then the mixture was centrifuged for 5 min. Finally, 200 mL of 10% HCl was applied twice to the collected solid material, followed by 200 mL of water for centrifugal separation/washing/redispersion. The produced precipitate was oven-dried overnight at 60 °C.

#### 2.2.2. Bismuth tungstate (Bi_2_WO_6_) preparation

The hydrothermal method was applied for the Bi_2_WO_6_ as follows; 2.91 g (6 mmol) of (Bi(NO_3_)_3_·5H_2_O) was dissolved in 37.5 mL of water. Subsequently, the solution was added to 12.5 mL of ethylene glycol containing 0.99 g (3 mmol) of Na_2_WO_4_. 2H_2_O. The mixture was transferred to a 75 mL Teflon-lined stainless-steel autoclave to be kept for 12 h at 160 °C. The resultant precipitates were centrifuged, washed several times with water, and dried for 12 h at 50 °C. The obtained sample was divided into parts and calcined at 700, 600, 500, and 400 °C for 4 h in air. The as-calcined products (BW-X) were named BW-IV, BW-III, BW-II, and BW-I, respectively.

#### 2.2.3. rGO/Bi_2_WO_6_ (GBW) preparation

**Fig. 1:**
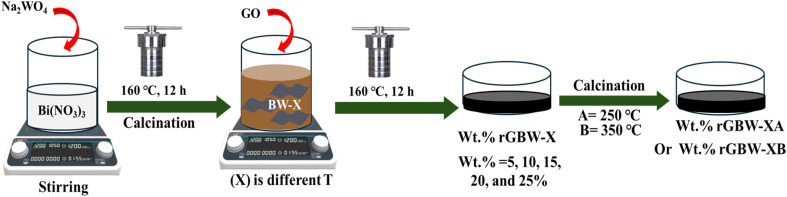
Scheme of synthesis of wt% rGBW calcined at different temperatures.

The Bi_2_WO_6_ and graphene oxide act as the starting materials for the preparation of rGO/Bi_2_WO_6_ composite. 1.0 g of Bi_2_WO_6_ was sonicated in 50 mL DI H_2_O, and the equivalent amounts of graphene oxide were suspended in DI H_2_O (20 mL) for 1 h, respectively. Then, the obtained brown solution of GO was added to the as-prepared Bi_2_WO_6_, dispersed by ultrasonication for 1 h and stirred for 6 h. The obtained solution was transferred to a 75 mL Teflon-lined stainless-steel autoclave to be kept at 160 °C for 12 h. The hydrothermal procedure is used for the reduction of GO to rGO.^59^ The resulting nanocomposites (rGBW) were centrifuged, washed, and dried in an oven at 80 °C. Meanwhile, certain amounts of GO were mixed with the Bi_2_WO_6_ at different calcination temperatures, and the composites (10rGBW-X) were named 10rGBW-I, 10rGBW-II, 10rGBW-III, and 10rGBW-IV. Then, these composites were calcined at 250 and 350 °C. These samples were named 10rGBW-IA, 10rGBW-IIA, 10rGBW-IIIA, and 10rGBW-IVA for samples calcined at 250 °C and 10rGBW-IB, 10rGBW-IIB, 10rGBW-IIIB, and 10rGBW-IVB for samples calcined at 350 °C. By the same procedures, the sample (BW-IV) was loaded with 5, 10, 15, 20 and 25% of GO and calcined at 250 °C and named 5rGBW-IVA, 10rGBW-IVA, 15rGBW-IVA, 20rGBW-IVA, and 25rGBW-IVA, respectively (Fig. 1).

### 2.3. Characterization

Powder X-ray diffraction (XRD) was carried out to detect the crystallinity of the materials utilising a Cu K radiation source, Ni filter, and PW150 (Philips). The FT-IR spectrum was recorded utilizing a Thermo SCIENTIFIC (NICOLET iS10) FTIR spectrometer after the dilution of each sample (2 mg) by 200 mg KBr. JEOL- Japan Transmission and Scanning Electron Microscopy with X-ray spectroscopy (EDS) were used for morphological evaluation and sample mapping. Using Ultraviolet-visible (UV-vis) diffuse reflectance spectroscopy, the band gaps of rGO/Bi_2_WO_6_ and pure Bi_2_WO_6_ photocatalyst samples were calculated using the absorbance and wavelength values for the samples. The Kubelka–Munk function, which is presented below, may be used to calculate the energy band gap value:^60^

1 *αhν* = (*hν* − *E*_g_)^*n*^

where *α*, *E*_g_, and *hν* are the absorption coefficient, the optical energy band gap value, and the photon energy (eV), respectively, and *n* = ½, 2, 3/2 and 3 are directly allowed, indirectly allowed, directly forbidden, and indirectly forbidden transitions, respectively. By utilizing the absorption data, the direct band gap value was calculated using Tauc's relation by projecting the linear plot of (*αhν*)^2^*versus hν* to the *x*-axis. The constant *n* is established by the semiconductor, which results in a direct band gap for *n* = ½. A PREVAC EA15 system equipped with a 180° electrostatic hemispherical analyser (HSA) was used to detect the X-ray photoelectron spectroscopy (XPS), applying a monochromatic Al K_α_ radiation, which is operated at 12 kV and 25 mA X-ray source. The concentrations of dyes were investigated before and after the photodegradation tests through a UV-vis absorption spectrophotometer. Finally, the electrochemical measurements were tested using a Corrtest potentiostat/galvanostat with a three-electrode configuration.

### 2.4. Photocatalytic degradation tests

The photocatalytic studies with the manufactured rGO/Bi_2_WO_6_ catalysts were carried out by evaluating the photodegradation rate of dyes under the influence of visible light. The studies were done under visible light irradiation (*λ* ≥ 420 nm) using a cut-off filter. The light intensity was calibrated with a photometer and constantly maintained at 100 mW cm^−2^ throughout the testing. Using DI water, stock solutions of RhB and MB (100 ppm) were prepared, and the required experimental concentrations of MB and RhB (5–50 ppm) were achieved by sequentially diluting the stock. The pH of MB and RhB practical solutions was changed with drop-wise additions of 0.1 M HCl/NaOH solutions. Pollutant solution (50.0 mL) was placed inside the photoreactor with a capacity of 100 mL. The photocatalyst was carefully inserted into the beaker containing the dye solution and was oriented horizontally at the reactor's centre and bottom. Before the photodegradation, the catalyst was suspended in a dye solution and was allowed to settle for 60 min to guarantee that the catalyst and dye solution were in equilibrium. A visible light source was placed around 10 cm above the reactor, and the degradation processes took place for three hours. The UV-vis spectrophotometer was utilized to investigate the change in pollutant concentrations at regular intervals. The following expression [eqn (2)], was used to determine the deterioration efficacy:^2^2
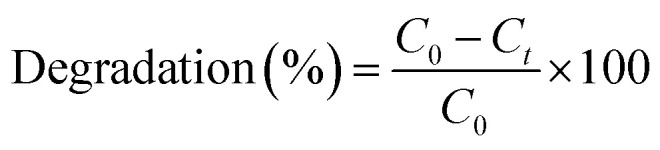
where *C*_o_ and *C*_*t*_ are the dye starting concentration (ppm) and at any given time (min), respectively.

### 2.5. PEC water splitting measurements

The photoelectrochemical tests were performed at room temperature on a Corrtest potentiostat equipped with a three-electrode system including a reference electrode (Ag/AgCl), counter electrode (Pt wire), and working electrode (indium tin oxide (ITO) glass substrate). According to the Nernst equation, the measured potentials were converted to the RHE scale as follows:

3 *E*_(RHE)_ = *E*(Ag/AgCl) + *E*°(Ag/AgCl) (0.197 V) + 0.059 × pH

The photocatalyst electrodes were prepared by dispersing 2 mg of each catalyst in 450 μL ethanol and 10 μL Nafion (5.0 wt%) as a binder under vigorous sonication for 1 h till complete homogeneity. After that, 40 μL of the homogenous dispersion was dropped on a well-cleaned ITO glass substrate (1 cm × 1 cm). Linear sweep voltammetry (LSV) was conducted in 1.0 M KOH electrolyte solutions with a scan rate of 5 mV s^−1^ under dark and light conditions. The photoresponse of the photocatalysts was measured in 1.0 M Na_2_SO_4_ solution under dark and light conditions. The electrochemical impedance spectroscopy (EIS) technique was applied from 100 kHz to 0.01 Hz.

## 3 Results and discussion

### 3.1. Structural characterization

**Fig. 2:**
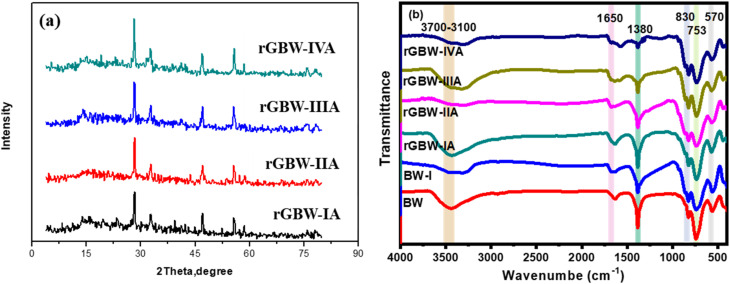
(a) XRD patterns of rGO doped Bi_2_WO_4_ at different calcination temperatures, and (b) FTIR spectra of BW, BW-1, 10rGBW-IA, 10rGBW-IIA, 10rGBW-IIIA and 10rGBW-IVA.

The phase structure of the produced catalysts was investigated by XRD analysis. Fig. S1a† depicts the XRD patterns for Bi_2_WO_6_ at different calinations. The orthorhombic phase of Bi_2_WO_6_ is characterized by several diffraction peaks at 2*θ* = 28.3, 32.7, 47.1, 55.8, 58.5, 68.7, 75.9, and 78.3° which are connected to the crystal planes of (131), (200), (202), (133), (262), (333), and (391), respectively.^61,62^ The crystal structure of Bi_2_WO_6_ was verified by observing narrow diffraction patterns when the calcination temperature was raised. The XRD patterns of the 10rGBW-IA, 10rGBW-IIA, 10rGBW-IIIA, and 10rGBW-IVA are shown in Fig. 2a, while Fig. S1b† displays the XRD patterns of rGBW-IVA with various amounts of rGO (5, 10, and 25%). The similar appearance of the diffraction peaks of Bi_2_WO_6_ and rGO/Bi_2_WO_6_ composites indicates the strong contact between the Bi_2_WO_6_ particles and rGO, which formed an interconnected structure. As rGO concentration rose, the diffraction peaks of Bi_2_WO_6_ were retained in the rGO/Bi_2_WO_6_ heterojunction, while their intensity eventually diminished. The average crystallite size can be calculated from the Debye–Scherrer formula using the peak with the highest intensity (2*θ* = 28.3°).

4 *D* = 0.94 *λ*/*β* cos *θ*

where *θ* is Bragg's angle, *λ* is the X-ray radiation wavelength, *D* is the average crystallite size, and *β* is the diffraction line widening as measured at the full-width half-maximum value (FWHM).

The crystallite size of Bi_2_WO_6_ increased from 20.5 to 49.9 nm with an increase in calcination temperature from 400 to 700 °C according to the Table 1. Likewise, rGO/Bi_2_WO_6_ composite demonstrates an increase in crystallite sizes when compared to pure Bi_2_WO_6_ formed at the same combustion temperature. It shows that after combining with reduced graphene oxide, the development of Bi_2_WO_6_ nanoparticles was constrained, which may be attributed to the confinement effect of rGO sheets. When Bi_2_WO_6_ particles are intercalated into stacked rGO layers, the stacking disorder causes the rGO diffraction peak to disappear in rGO/Bi_2_WO_6_ hybrids.

**Table 1:** The crystal sizes and band gaps of Bi_2_WO_6_ and rGO/Bi_2_WO_6_ samples

Catalyst	Crystallite size (nm)	Band gap (eV)	Catalyst	Band gap (eV)	Crystallite size (nm)
BW-I	20.5	2.65	10rGBW-IA	2.63	27.4
BW-II	25.3	2.59	10rGBW-IIA	2.54	31.5
BW-III	36.9	2.37	10rGBW-IIIA	2.29	45.1
BW-IV	49.9	1.72	10rGBW-IVA	1.41	61.2
—	—	—	5rGBW-IVA	—	54.6
—	—		25rGBW-IVA		69.5

The FT-IR spectra of BW and BW-I (Fig. 2b) show the main absorption bands, which are related to the W–O–W bridging stretching, Bi–O, W–O, and W–O–C stretching modes at 900–400 cm^−1^.^63^ Bi_2_WO_6_ and rGO in the composite are chemically interconnected, which is confirmed by W–O–C stretching vibrations.^64,65^ The stretching of Bi–O was ascribed to the FTIR spectra obtained at 451 cm^−1^, while the peak at 554 cm^−1^ is ascribed to Bi–O polyhedral bending vibration and WO_6_ bending vibration and stretching.^29^ The absorption peaks at 815–860 cm^−1^ are due to the bridge stretching vibration of W–O in WO_6_ octahedral, whereas the peaks at 734 cm^−1^ are related to the stretching vibration of WO_6_ in Bi_2_WO_6_.^12^ The typical FTIR spectra of rGO/Bi_2_WO_6_ display distinctive bands of the different types of carbon–oxygen interaction, including C–O alkoxy (1036 cm^−1^), C–O epoxy (1222 cm^−1^), C–O stretching (1378 cm^−1^), and C=O carbonyl (1640 cm^−1^). At 3434 cm^−1^, a wide band appeared, which is indicative of the –OH group.^52,66^ Conversely, the oxygen-containing vibrational bands in the spectra of the rGO/Bi_2_WO_6_ composite were dramatically decreased with high calcination temperatures, confirming a considerable reduction of GO during hydrothermal treatment. Once the composite was developed, the wide band at 3430 cm^−1^ corresponding to the stretching and bending vibration of –OH and that of C=O carbonyl at 1640 cm^−1^ were much diminished and nearly eliminated. The results presented here confirm that GO was successfully reduced to rGO and that the rGO/Bi_2_WO_6_ composite was formed.

The linear relation of (*αhν*)^2^*versus* (*hν*) for Bi_2_WO_6_ calcined at 400, 500, 600, and 700 °C is shown in Fig. S2a,† and that for BW-IV, 10rGBW-IA, 10rGBW-IIA, 10rGBW-IIIA, and 10rGBW-IIVA nanocomposites are illustrated in Fig. S2b.†Table 1 findings demonstrated that the band gaps of rGBW-IVA and BW-IV had the lowest band gap energy values. The absorbance has therefore moved from the ultraviolet to the visible range as a result of the band gap being decreased. More photogenerated e^−^/h^+^ couples at the redshift suggest that photons with lower energy may ignite them, improving photocatalytic degradation efficiency in the visible spectrum.^2^ The synthesized xrGBW-IVA nanocomposites were shown to have a smaller band gap in all cases, confirming a potential improvement in photocatalytic activity. Table 1 further shows that the band gap values decrease as the crystallite size of the produced samples increases. The reduced bandgap is beneficial for increasing the adsorption of light for rGO/BiWO_4_ composites, resulting in improved photocatalytic activity.^67^

**Fig. 3:**
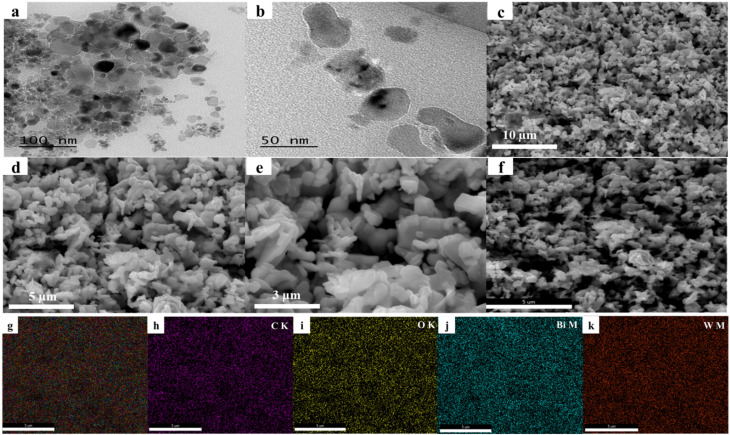
(a and b) TEM images, (c–e) SEM images, and (f and g) EDX mapping of 10rGBW-IVA, and mapping images of (h) C, (i) O, (j) Bi, and (k) W.

From TEM images of 10rGBW-VIA (Fig. 3a and b), the as-prepared Bi_2_WO_6_ are depicted as roughly spherical particles with a small average size of 30 nm, which are dispersed on the rGO surface. Additionally, the Bi_2_WO_6_ particles can be seen to be equally scattered across the graphene sheets and to be shaped like elongated, uneven flakes.^16,68^ The TEM examination further showed that the overlapping of rGO sheets on the Bi_2_WO_6_ particles created a three-dimensional lattice structure that facilitated quicker electron mobilization in visible light. Moreover, the SEM morphology of the produced 10rGBW-VIA sample is shown in Fig. 3c–e. The figure showed that the particle sizes of Bi_2_WO_6_ are transformed into a significant number of spherical-like and pseudo-tetragonal particles that are evenly dispersed across graphene.^69^ According to the data above, the connection between Bi_2_WO_6_ and rGO particles was enhanced by the calcination procedure. Also, Fig. 3f shows the SEM image of the 10rGBW-VIA composite used for EDX elemental mapping. The illustration shows a very homogeneous and aggregated surface structure, which is appropriate for elemental analysis. Fig. 3g–k shows the elemental distribution of the synthesised rGO/Bi_2_WO_6_ composite analysed using EDX mapping. The mapping image (Fig. 3g) confirms that all key elements present in the composite are uniformly distributed. Fig. 3h displays the carbon (C) signal, which originates from the reduced graphene oxide (rGO) sheets, proving their homogeneous incorporation throughout the sample. The consistent dispersion of oxygen (O) (Fig. 3i), bismuth (Bi) (Fig. 3j), and tungsten (W) (Fig. 3k) indicates the even distribution of Bi_2_WO_6_ nanoparticles on the rGO matrix. The absence of element clustering or segregation indicates good mixing and robust interfacial contact between rGO and Bi_2_WO_6_, which is crucial for efficient charge transfer and separation. Besides, the chemical composition of the 10rGBW-VIA nanoparticles was investigated using EDX analysis, as shown in Fig. S3.† The sample components were identified *via* EDX analysis, which revealed the presence of C, W, Bi, and O. As a result, the rGO/Bi_2_WO_6_ nanocomposite was successfully synthesized, as shown by the SEM and EDX mapping.

**Fig. 4:**
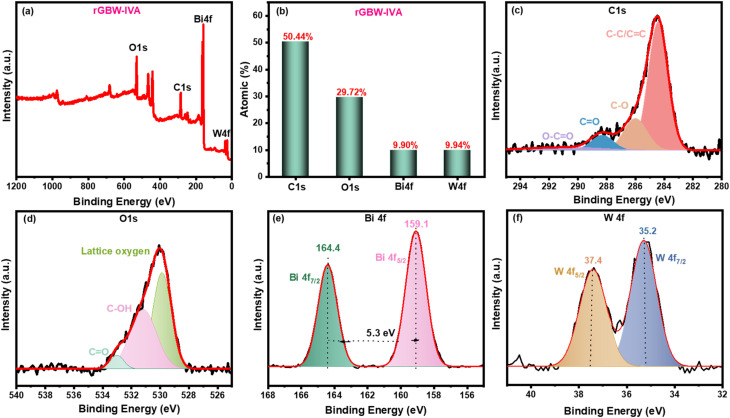
XPS of 10rGBW-IVA, (a) Total survey spectra, (b) atomic % of each component, and core-level spectra of (c) C 1s, (d) O 1s, (e) Bi 4f, and (f) W 4f.

XPS analysis was applied to identify the chemical composition on the surface of rGBW-IVA. Fig. 4a and b shows the atomic percentage of each component and the overall XPS survey. Four peaks in the whole XPS spectrum are seen at 533, 285, 159, and 38 eV. These are ascribed to O 1s, C 1s, Bi 4f, and W 4f, in that order. It was found that the atomic percentages of O 1s, C 1s, Bi 4f, and W 4f were, in order, 29.72, 50.44, 9.90, and 9.94%. Four Gaussian peaks are identified with binding energies of 284.4, 286.1, 288.2, and 289.5 eV in the XPS spectra of C 1s (Fig. 4c). For the non-oxygenated carbon of C–C/C–H in the rGO structure, the characteristic peak appeared at 284.4 eV. Moreover, the Gaussian peak at 286.1 eV represented the hydroxyl or epoxide (C–OH/C–O) functional groups. The carbon functionalized in the carbonyl group appeared at 288.2 eV. Moreover, the low peak intensity at 289.5 eV, which is related to the carboxylic group, confirmed the reduction of the GO to rGO.^70,71^ Comparably, three Gaussian peaks can be seen in the XPS spectrum of O 1s (Fig. 4d). The peak appearing at 529.8 eV is characteristic of lattice oxygen in Bi_2_WO_6_. While the peaks at 531.2 and 533.0 eV are assigned to C–OH and C=O, respectively.^72,73^ Additionally, two Gaussian peaks are observed in the Bi 4f spectrum (Fig. 4e) at 164.4 and 159.1 eV, which are related to Bi 4f_5/2_ and Bi 4f_7/2_ spin–orbital splitting photoelectrons in anatase Bi_2_O_3_, respectively. These results reveal the presence of trivalent oxidation state for bismuth (Bi^3+^) as it occurs in the Bi_2_WO_6_ hollow structure.^74^ Ultimately, oxidized W ions are related to the W 4f_5/2_ and W 4f_7/2_ spin–orbital splitting in WO_3_, which are situated at 37.5 and 35.2 eV, respectively, confirming a hexavalent oxidation state of tungsten (W^6+^).^75^

Fig. S4a† depicts a nitrogen adsorption–desorption isotherm of 10rGBW-IVA with a Type IV profile and a significant hysteresis loop, indicating mesoporous materials according to the IUPAC classification. The nitrogen adsorption gradually increases with rising relative pressure (*P*/*P*^0^), followed by a sharp surge around *P*/*P*^0^ = 1.0, due to capillary condensation within mesopores.^76^ Moreover, the H_3_-type hysteresis loop in the curve indicates the presence of slit-like pores or the formation of plate-like particles. Fig. S4b† depicts the pore size distribution obtained using the BJH (Barrett–Joyner–Halenda) method, which displays a prominent pore size in the range of 2–5 nm, confirming the mesoporous nature of 10rGBW-IVA. These findings collectively confirm the formation of ultra-thin nanosheet structures, increasing the material's BET surface area and creating more active sites for photocatalytic activity.

### 3.2. The photocatalytic degradation of dyes

#### 3.2.1. Effects of calcination temperatures and GO content

**Fig. 5:**
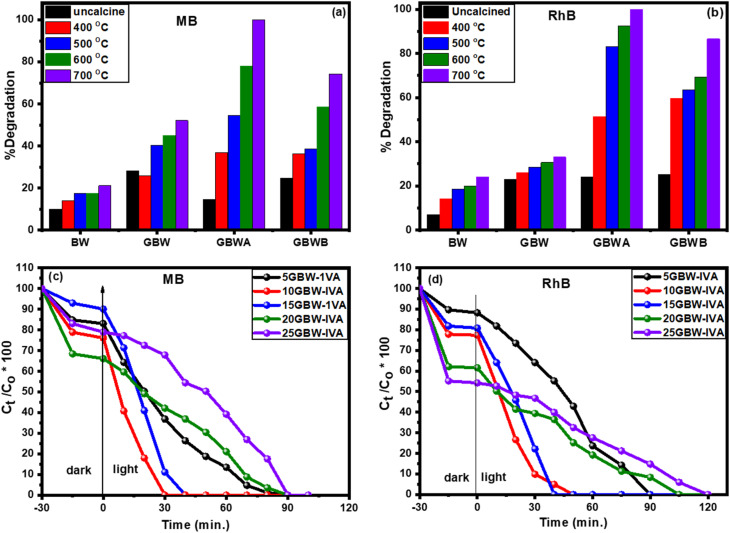
% Degradation of (a) MB and (b) RhB at different calcination temperatures, and degradation of (c) MB and (d) RhB with different amounts of GO.

Under visible light irradiation, the influence of the calcination temperature of BW and GBW on photocatalytic degradation of MB and RhB was performed to detect the optimum conditions. For pure BW, the experiments revealed that the increase in calcination temperature from 400 to 700 °C increased the photodegradation. The percentage degradation after 240 min was about 10.3, 19.4, 14.9, 16.6, and 19.4% (Fig. 5a and S5a†) for MB and 13.7, 22.3, 25.2, 29.2, and 30.3% for RhB (Fig. 5b and S5b†) using BW, BW-I, BW-II, BW-III, and BW-IV, respectively. These results indicated that BW-IV is the best photocatalyst from all samples. In Fig. 5a, b, S6a and b,† a noticeable improvement in the degradation of dyes was observed after the addition of GO. After 240 min, MB degradation was about 19.3, 21.6, 23.9, 28.5, and 33.2% and RhB was 9.1, 18.9, 21.8, 24.7, and 28.4% for GBW, GBW-I, GBW-II, GBW-III, and GBW-IV, respectively. The highest value of MB and RhB dyes was observed by the usage of GBW-IV. This value may be due to the low band gap related to the rise in the temperature of calcination and the presence of GO. On the other hand, the photocatalysts of GBW-A, GBW-IA, GBW-IIA, GBW-IIIA, and GBW-IVA in Fig. 5a, b, S7a and b† exhibited higher photodegradation due to the calcination effect. After 60 min, the percentage removal of MB and RhB reached up to 97.1 and 91.1%, respectively, using the GBW-IVA catalyst. Also, Fig. 5a, b, S8a and b† show a significant decrease in the photocatalytic degradation of MB (89.3%) and RhB (72.1%) by the GBW-IVB catalyst. The results indicated that the sample BW-IV (calcination at 700 °C) is the most suitable temperature for the calcination of Bi_2_WO_6_. It reached the maximum, maybe because of the complete crystallization of Bi_2_WO_6_ at this temperature. Furthermore, the calcination of GBW-IVA causes the deoxygenation of the GO sheets, which leads to the complete reduction of GO to rGO with a low band gap and conductivity. The conductivity leads to high performance in anti-recombination and high-performance charge separation during the photocatalytic process. Also, graphene can play a serious role in inhibiting the aggregation of Bi_2_WO_6_ particles during thermal treatment. More information on the photodegradation of MB and RhB is listed in the Table S1,† respectively.

On the other hand, the amount of graphene is a crucial parameter in determining the photocatalytic activity of GBW-IVA nanocomposites. Fig. 5c and d show the effect of photodegradation of MB and RhB under visible light using GBW-IVA with different GO amounts as photocatalysts. After 30 min, the photocatalytic degradation increased with increasing the amount of GO, reaching up to 10 wt% GO in Bi_2_WO_6_ (Fig. S9†) and decreased with further increase of GO content. It has been concluded that the GO addition boosted the photocatalytic activities of Bi_2_WO_6_ because GO can promote the separation of electron holes and increase surface area during the reaction. The further concentration of graphene oxide increased the scattering and absorbance of photons, thus weakening the ability of photon absorption.

#### 3.2.2. Effect of pH on MB and RhB photodegradation

**Fig. 6:**
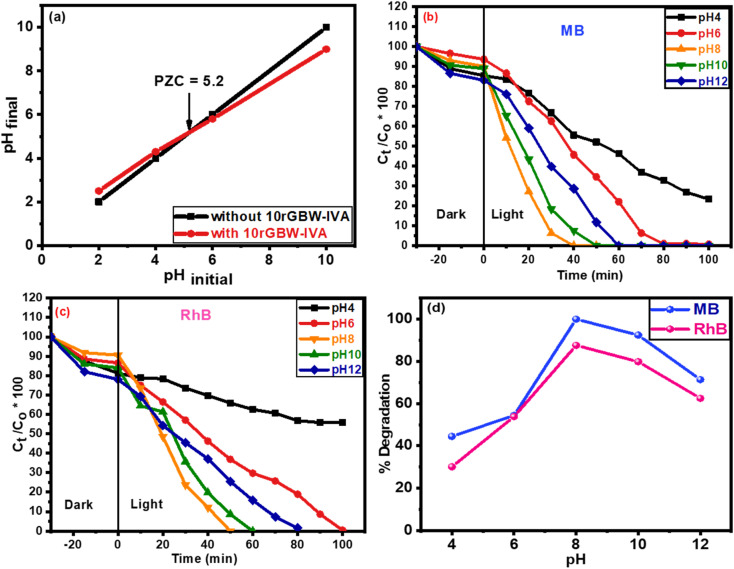
(a) Point of zero charges (PZC) measurements of MB dye with and without 10rGBW-IVA, irradiation time for (b) MB and (c) RhB at different pH values, and (d) percentage of dye degradation after 40 min of light irradiation at various pH values.

The point of zero charge (PZC) is the pH value at which the net charge of the sample surface is zero. Understanding the PZC value is crucial to comprehending the interactions that arise at the surface when pH varies. To compute the PZC, four bottles containing 50 millilitre solutions at pH values of 2.0, 4.0, 6.0, and 10.0 were created using 0.1 M HCl and 0.1 M NaOH. At room temperature, each solution was mixed with 100 mg of 10GBW-IVA and the mixture was shaken for 48 hours. Plotting the final pH against the initial pH (2.0, 4.0, 6.0, and 10.0), as shown in Fig. 6a, reveals that the PZC is at pH = 5.2, where the photocatalyst surface is almost neutral. Hydrogen ions are adsorbed on the catalyst active sites and become positively charged when the pH falls below 5.2. When the pH value increases up to 5.2, the catalyst surface will have more hydroxide ions and progressively transition to a negatively charged state.^77^

The pH of the MB and RhB solutions was changed from 4.0 to 12.0 to optimize the parameters of the reaction. The test was performed with a catalyst dose of 75 mg, a degradation time of 40 min, and an initial dye concentration of 10 ppm. As illustrated in Fig. 6b and c, the breakdown efficiency of MB rose from 44.5% at pH 4.0 to 54.4% at pH 6.0, eventually reaching 100% at pH 8.0. Similarly, RhB degradation increased from 30.1% to 87.6% within the same pH range. However, subsequent pH increases to 10.0 and 12.0 resulted in a decrease in breakdown efficiency (Fig. 6d). This behaviour is due to electrostatic interactions between the dye molecules and the photocatalyst.^78^ At moderately basic pH, the rGBW surface becomes negatively charged, which promotes cationic dye adsorption (MB and RhB) *via* electrostatic attraction. Although ˙OH radical formation increases at high pH levels, the degradation rate reduces, which could be due to radical quenching or dye desorption. In contrast, under acidic circumstances, the positively charged photocatalyst surface repels cationic dyes, reducing their adsorption and degradation.^79^

#### 3.2.3. Effect of initial concentrations and catalyst dosage

The impacts of starting concentrations of dyes on the photocatalytic performance were examined. The initial concentrations = ranged from 5 to 50 ppm with 75 mg of 10rGBW-IVA and solution pH = 8. The degradation efficiencies of dyes decrease as the concentration of dye increases, as seen in Fig. S10(a–c).† This could be because active areas on rGBW-IVA are more accessible at lower dye concentrations, which improves adsorption, promotes dye excitation, and intersystem crossover. At greater dye concentrations, degradation slows, possibly due to active site blocking by excess dye molecules, which inhibits ˙OH radical production and increases light shielding.

On the other hand, the influence of the catalyst dosage on the MB and RhB photodegradation was investigated using different amounts of 10rGBW-IVA (Fig. S10d†). Because of the presence of more active sites on the surface of the 10rGBW-IVA, the increasing catalyst dosage boosted photocatalytic activity. However, photodegradation of the catalyst was reduced when the catalyst dose surpassed 75 mg. This leads to increased production of ˙OH and O_2_^−^ radicals, which improve pollutant breakdown. However, efficiency decreases after an optimal catalyst dose due to increased turbidity and light scattering, which limit radiation penetration. Furthermore, particle agglomeration at high concentrations decreases the active surface area available for photocatalysis.

#### 3.2.4. Kinetic studies, reusability of catalyst and mechanism of photodegradation

**Fig. 7:**
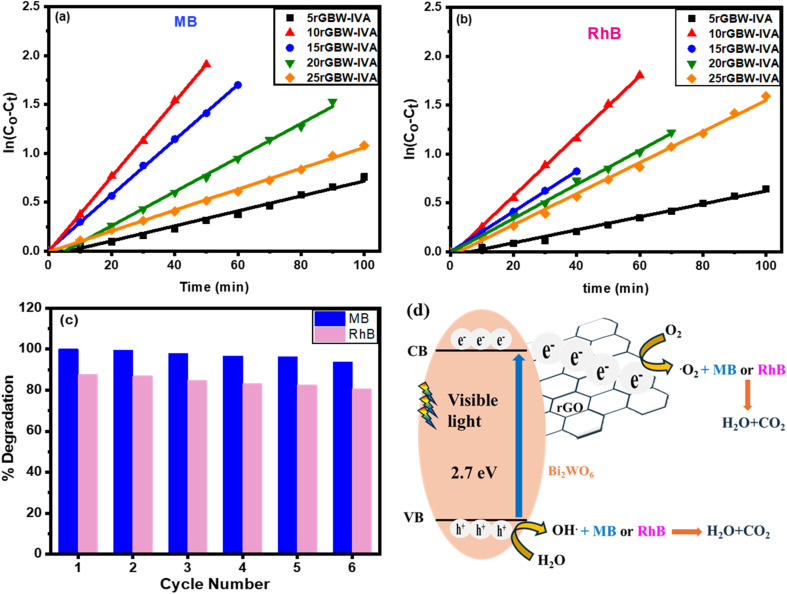
First-order kinetics of (a) MB and (b) RhB degradation over rGOBW-IVA at various amounts of rGO content, (c) photodegradation recycling for RhB and MB of 15rGOBW-IVA nanocomposite under visible light irradiation, and (d) diagram illustrating how rGO reduces the Bi_2_WO_6_ conduction band potential.

Kinetic tests on the MB and RhB photodegradation were performed to validate the kinetic model and calculate the reaction rate constant values. The study was performed at pH = 8.0 under visible light irradiation utilizing xrGBW-IVA photocatalysts. The entire photodegradation kinetics is governed by a pseudo-first-order of the Langmuir–Hinshelwood (L–H) mechanism, which has been demonstrated for photocatalysis at low initial concentrations of MB and RhB dyes. The following is the pertinent equation (eqn (5)):

5 Ln(*C*_o_ − *C*_t_) = *k*_app_*t*

while (*C*_o_ − *C*_*t*_) represents the decrease in dye initial concentrations after different time intervals, and the first-order rate constant (s^−1^) is denoted by *k*_app_. The *k*_app_ values are now calculated using the plot of ln(*C*_o_ − *C*_*t*_) as a function of time. The linear relationship of pseudo-first-order kinetics is observed in Fig. 7a and b. Also, Table S2† displays the pseudo-first-order rate constant and *R*^2^ values when rGO content and dye concentration are altered. The results show that the pseudo-first-order rate kinetics are suitable for dye degradation. The rise in rGO content causes an increase in the rate constant values, reaching the maximum value using 10rGBW-IVA. Using 15rGBW-IVA, the photodegradation of MB and RhB at various initial concentrations followed pseudo-first-order kinetics under visible light, which was consistent with the observed activity data. Table S2† further shows that when the initial dye concentrations grow, the *k*_app_ decreases.

The reusability of the photocatalyst is necessary to evaluate the long-term stability of the rGOBWIV nanocomposite. The catalyst was subjected to 6 cycles of degradation efficiency assessment of MB, as shown in Fig. 7c. The results indicated that the 10rGBW-IVA catalyst could preserve an average degradation efficiency of 97.1% and 84.7% for MB and RhB, respectively. These results indicated the potential reusability of the samples. The trivial lowering of the activity may be due to powder loss during recycling.

When the photocatalyst is irradiated, the formation of ROS, such as superoxide or hydroxyl radicals, is the key factor in the photomineralization of organic dyes. Photocatalytic performance under visible light can be attributed to defect production and the narrowing of the energy bandgap caused by the formation of the rGO/Bi_2_WO_6_ heterostructures. Fig. 7d shows the photocatalytic mechanism of MB and RhB using the GO-based Bi_2_WO_6_ catalyst. When Bi_2_WO_6_ is exposed to UV and visible light, electrons (e^−^) from the VB are excited to the CB and leave holes (h^+^) in the VB, as explained in (6). Since the work function of rGO is less than that of the CB of the Bi_2_WO_6_, the e^−^(CB) can easily transfer to the GO surface (7). These electrons can react with oxygen to generate superoxide radicals and hydroxyl radicals (8). Moreover, the holes in VB can react with hydroxide ions (9) and water (10) to form hydroxyl radicals. Finally, the photogenerated radicals can oxidize organic dyes (11) in an oxidation process, resulting in the formation of H_2_O and CO_2_. The Fermi level shift and conduction band potential drop caused by the charge equilibration and electronic contact between graphene and Bi_2_WO_6_ have a significant impact on the process of photocatalytic conversion.

6 rGO/Bi_2_WO_6_ + *hν* → rGO/Bi_2_WO_6_ (h^+^ (VB) + e^−^ (CB))

7 rGO/Bi_2_WO_6_ (h^+^ + e^−^) → rGO(e^−^) + Bi_2_WO_6_ (h^+^)

8
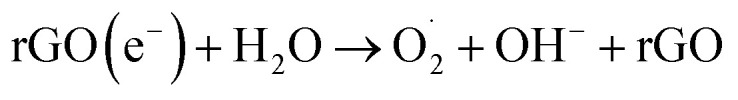

9 Bi_2_WO_6_ (h^+^) + OH^−^ → Bi_2_WO_6_ + ˙OH

10 Bi_2_WO_6_ (h^+^) + H_2_O → Bi_2_WO_6_ + H^+^ + ˙OH

11 MB or RhB + ROS → CO_2_ +H_2_O

On the other hand, scavenger studies with specific quenching agents were carried out to examine the underlying photocatalytic mechanism and determine the major reactive species. Scavengers such as isopropyl alcohol (IPA), benzoquinone (BQ), and disodium ethylenediaminetetraacetate (EDTA-2Na) were utilized to trap HO˙, 
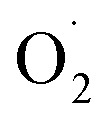
, and holes produced during photo-oxidation, respectively. Fig. S11† shows that after the addition of EDTA, the photocatalytic reaction capacity of 10 rGBW-IVA towards MB dye was significantly reduced by 17.39% when compared to the composite without scavenger. When BQ and IPA were introduced to the reaction system, the MB degradation efficiency was lowered by 47.08% and 51.87%, indicating that these species were actively involved in the MB photodegradation process.

### 3.3. Photoelectrochemical water splitting

**Fig. 8:**
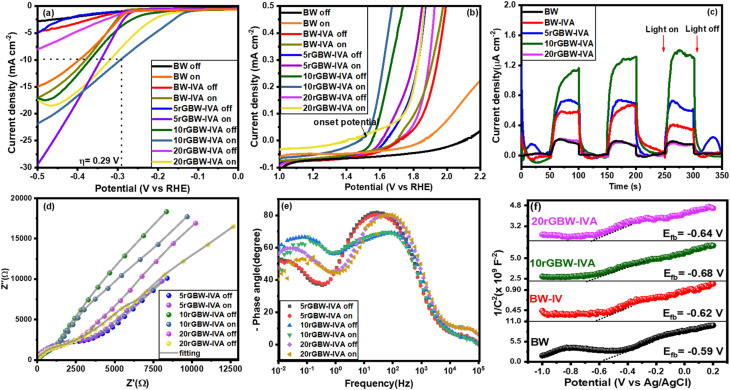
(a and b) LSV curve for HER and OER activities, (c) chronoamperometry technique, (d) EIS spectrum, (e) Bode plot, and (f) Mott–Schottky curve.

The as-prepared photocatalysts were tested in the PEC water splitting to investigate their photoelectrochemical properties. The photocatalysts with different amounts of rGO were tested on the PEC oxygen and hydrogen evolution from water splitting. These experiments were conducted by employing a three-electrode configuration system in 1 M KOH (pH = 13.22) under both dark and illuminated conditions, using a simulated solar spectrum (100 mW cm^−2^) as the light source. Fig. 8a represents the LSV of all samples against electrochemical hydrogen evolution reaction (HER) under dark and light conditions. Compared to the bare BW, the LSV curves of the calcined BW and rGO composites exhibited improved HER performance. Moreover, the activities of these samples further increased under illuminated conditions compared to those observed in the dark. At 10 mA cm^−2^ current density, the 10rGBW-IVA electrode exhibited an overpotential of 290 mV, surpassing those of other electrodes in the dark or under light. On the other hand, Fig. 8b represents the LSV of all samples against electrochemical oxygen evolution reaction (OER) under dark and light conditions. The figure shows that the onset potential of the OER shifted to more negative potentials after the calcination and combination of BW with rGO. It was observed that 10rGBW-IVA showed more negative onset potentials and higher current density under dark conditions due to its low band gap. On the other hand, all samples showed more negative onset potentials and high current densities under solar illumination compared to dark conditions. The onset potential was higher in dark conditions compared to light conditions, suggesting an enhancement in performance when transitioning from dark to light. Achieving low onset potential requires materials with suitable surface features that facilitate the efficient transfer of charges. This surface characteristic has an important role in investigating the catalytic performance of a material.^80^ The 10rGBW-IVA catalyst showed an onset potential of 1.53 V *vs.* RHE with a high current density of 35 mA cm^−2^.

Furthermore, the chronoamperometric technique (*I*–*t* curve), with three cycles of a 50-s interval light on or off, was also applied to investigate the photoresponse of different materials. Fig. 8c confirms the high photoresponse of the 10rGBW-IVA electrode (∼1.4 μA cm^−2^) and the high stability and repeatability of the as-synthesised photoanodes. The observed results show a considerable performance improvement when compared to similar photocatalytic materials described in the literature (Table S3†). The improved photoresponse is due to a synergistic interaction between graphene oxide (GO) and bismuth tungstate, which allows for more effective separation and transmission of photogenerated electron–hole pairs. Fig. 8d displays the photoelectrochemical impedance spectra produced during the experiment, as well as the fitted equivalent circuit model used to interpret the data. This model gives information about the charge transfer processes and resistances in the system. EIS results were obtained using an AC voltage (0 V) amplitude of 20 mV and a frequency sweep from 0.1 to 10^5^ Hz, under both dark and illuminated conditions. The EIS measurements are used to generate the Nyquist plot, which provides insight into the reaction rate at the surface of the electrode through the radius of the semicircle observed in the plot. The Nyquist plot suggests that the 10rGBW-IVA photocatalyst exhibits a smaller arc radius in both dark and light conditions, confirming a lower resistance of charge transfer and potentially improved performance. The narrower arc radius of the Nyquist plot indicated the lower charge transfer resistance, which leads to increased conductivity and better separation of photogenerated e^−^/h^+^ pairs at the electrode/electrolyte interface, hence increasing photocatalytic efficiency.

The Bode plot of the EIS technique was used to calculate the relaxation time of photogenerated electrons. As observed in Fig. 8e, the lifetime (*τ*) of photoelectrons was estimated from the maximum peak (*f*_max_) of the Bod plot using the following equation:^81,82^

12 *τ* = 1/(2π *f*_max_)

The highest peak in the low-frequency band correlates with the photoelectrons' lifetime. The corresponding lifetime of electrons is found to be 6.01, 1.86, and 2.61 ms for 5rGBW-IVA, 10rGBW-IVA, and 20rGBW-IVA, respectively. This extended lifetime suggests that the photocatalyst facilitates photogenerated electron transfer, thereby mitigating electron–hole recombination. Moreover, the flat band (FB) potentials were evaluated for the as-prepared photoanodes from the Mott–Schottky analysis. Taken from the interruption of the plot with *X*-axis (Fig. 8f), the potentials of FB are found to be −0.59, −0.62, −0.68, and −0.64 V *vs.* Ag/AgCl (0.01, −0.02, −0.08, and −0.04 V *vs.* RHE) at pH = 7 for BW, BW-IV, 10rGBW-IVA, and 20rGBW-IVA, respectively. The positive slopes of the curves suggested that all synthesised catalysts were n-type semiconductors, which have FB approximately close to their Fermi level. The stronger negative shift in the Fermi level for BW-based materials indicates a larger water-splitting oxidation potential, which is consistent with previous research.^83^

## 4 Conclusion

In summary, we effectively produced rGO/Bi_2_WO_6_ composites using a straightforward method that did not need any surface modification of the Bi_2_WO_6_ nanoparticles. Many analytical and spectroscopic approaches were used to characterise the synthesised solar light-driven nanocomposites. The resultant rGO/Bi_2_WO_6_ composites had improved photocatalytic activity and visible light absorption. The photocatalytic efficiency of 10% rGO/Bi_2_WO_6_ composites for the breakdown of MB and RhB is nearly 5 times that of pure Bi_2_WO_6_. The quick photo-generated rGO/Bi_2_WO_6_ photocatalytic activity can be attributed to the improved photocatalytic performance. In addition, the effects of catalyst loading, starting substrate concentration, rGO content, and reaction pH were investigated. Electrochemical impedance spectroscopy (EIS) and chronoamperometry techniques were used to evaluate the electrochemical characteristics of the synthesised materials. According to the findings, 10rGBW-IVA has the lowest charge-transfer resistance and the largest photoresponse. The improved photocatalytic activity of 10rGBW-IVA is due to the synergistic action between rGO and Bi_2_WO_6_ that induces the separation of photo-generated charge carriers. Based on these observations, a viable degradation pathway for MB and RhB dyes was proposed. The as-prepared GBW-based catalysts exhibit high catalytic activity for photoelectrochemical water splitting in alkaline aqueous.

## Conflicts of interest

The authors declared no potential conflicts of interest with respect to the research, authorship, and/or publication of this article.

## Supplementary Material

RA-015-D5RA04049C-s001

## Data Availability

All data underlying the results are available as part of the article and no additional source data is required.

## References

[cit1] Lim H., Yusuf M., Song S., Park S., Park K. H. (2021). Efficient photocatalytic degradation of dyes using photo-deposited Ag nanoparticles on ZnO structures: simple morphological control of ZnO. RSC Adv..

[cit2] Ahmed A. I., Kospa D. A., Gamal S., Samra S. E., Salah A. A., El-Hakam S. A., Awad Ibrahim A. (2022). Fast and simple fabrication of reduced graphene oxide-zinc tungstate nanocomposite with enhanced photoresponse properties as a highly efficient indirect sunlight driven photocatalyst and antibacterial agent. J. Photochem. Photobiol., A.

[cit3] Khan I., Saeed K., Zekker I., Zhang B., Hendi A. H., Ahmad A., Ahmad S., Zada N., Ahmad H., Shah L. A., Shah T., Khan I. (2022). Review on methylene blue: its properties, uses, toxicity and photodegradation. Water.

[cit4] Markandeya, Mohan D., Shukla S. P. (2022). Hazardous consequences of textile mill effluents on soil and their remediation approaches. Clean Eng. Technol..

[cit5] Negi A. (2025). Environmental Impact of Textile Materials: Challenges in Fiber-Dye Chemistry and Implication of Microbial Biodegradation. Polymers.

[cit6] Li Q., Zhou H., Zhang F., Yuan J., Dong D., Zhang L., Du L. (2024). Electrochemical treatment of malachite green dye wastewater by pulse three-dimensional electrode method. Environ. Technol..

[cit7] Li P., Zhang J., Yu Y., Jia W., Zhao S. (2024). A collaborative coagulation strategy for algae-laden and dye-containing water treatment. J. Clean. Prod..

[cit8] Bose P., Dash S. R., Kim J. (2025). Conductive CuO-CNT/PES membranes for electrochemical membrane filtration and advanced wastewater treatment. J. Water Proc. Eng..

[cit9] Duan Y., Sun S.-Y., Zhao J., Yuan H. (2025). Microplastics affect the removal of dye in textile wastewater: Adsorption capacity and its effect on coagulation behavior. Sep. Purif. Technol..

[cit10] Modi A., Baranda P., Thakor R., Thacker D., Trivedi J., Bariya H. (2025). Fungal consortium mediated efficient biodegradation of hazardous reactive dyes from textile effluent: An environmentally acceptable strategy. J. Hazard. Mater. Adv..

[cit11] Mylsamy S., Govindasamy T., Subramanian B. (2025). Defect-rich 3D ZnO/2D rGO nanocomposites: Insight into photocatalysis and photoelectrochemical water splitting. Mater. Today Chem..

[cit12] Song Y., Zhou F., Chai Y., Zhan S. (2021). Study on high antibacterial RGO/Bi2WO6 microspheres combined with PEVE coating for marine sterilization under visible light. Res. Chem. Intermed..

[cit13] Jin J., Dai C., Zeng C., Liu X., Jia Y. (2024). Bimetallic Au/Ag coated on In2O3 for the effective removal of emerging organic contaminants under natural sunlight irradiation. J. Environ. Manage..

[cit14] Sun M., Ali S., Liu C., Dai C., Liu X., Zeng C. (2024). Synergistic effect of Fe doping and oxygen vacancy in AgIO3 for effectively degrading organic pollutants under natural sunlight. Environ. Pollut..

[cit15] Jin J., Liu C., Dai C., Zeng C., Jia Y., Liu X. (2024). Boosting the activity for organic pollutants removal of In2O3 by loading Ag particles under natural sunlight irradiation. Environ. Res..

[cit16] Jaswal A., Kaur M., Kaur M., Kansal S. K. (2021). rGO-Bi2MoO6 heterostructure: synthesis, characterization and utilization as a visible light active photocatalyst for the degradation of tetracycline. J. Mater. Sci.: Mater. Electron..

[cit17] Kaid M. M., Elbanna O., El-Hakam S. A., El-Kaderi H. M., Ibrahim A. A. (2022). Effective photocatalytic degradation of organic dyes using ZNC/rGO nanocomposite photocatalyst derived from ZIF-8/rGO thermolysis for water treatment. J. Photochem. Photobiol., A.

[cit18] Rafiq A., Ikram M., Ali S., Niaz F., Khan M., Khan Q., Maqbool M. (2021). Photocatalytic degradation of dyes using semiconductor photocatalysts to clean industrial water pollution. J. Ind. Eng. Chem..

[cit19] Harris-Lee T. R., Marken F., Bentley C. L., Zhang J., Johnson A. L. (2023). A chemist's guide to photoelectrode development for water splitting – the importance of molecular precursor design. EES Catal..

[cit20] Goodarzi N., Ashrafi-Peyman Z., Khani E., Moshfegh A. Z. (2023). Recent progress on semiconductor heterogeneous photocatalysts in clean energy production and environmental remediation. Catalysts.

[cit21] Ren H., Miao Z., Zhao Y., Ghasemi S., Feng X., Liu E., Padervand M. (2025). Advances and challenges in multiple S-scheme heterojunction photocatalysts. J. Alloys Compd..

[cit22] Bashir S., Jamil A., Alazmi A., Khan M. S., Alsafari I. A., Shahid M. (2023). Synergistic effects of doping, composite formation, and nanotechnology to enhance the photocatalytic activities of semiconductive materials. Opt. Mater..

[cit23] Ishfaq M., Hassan W., Sabir M., Somaily H. H., Hachim S. K., Kadhim Z. J., Lafta H. A., Alnassar Y. S., Rheima A. M., Ejaz S. R., Aadil M. (2022). Wet-chemical synthesis of ZnO/CdO/CeO2 heterostructure: A novel material for environmental remediation application. Ceram. Int..

[cit24] Abo El-Yazeed W. S., El-Hakam S. A., Salah A. A., Ibrahim A. A. (2021). Fabrication and characterization of reduced graphene-BiVO4 nanocomposites for enhancing visible light photocatalytic and antibacterial activity. J. Photochem. Photobiol., A.

[cit25] Abo Kamar S. M., Ibrahim A. A., El-Hakam S. A., El-Sharkawy E. A., Ahmed A. I., Adly M. S. (2024). Architecture of interconnected cubic NiCo2S4 decorated mesoporous carbon with self-doped nitrogen based-hydrogel for high performance hybrid supercapacitor. J. Energy Storage.

[cit26] Athar M., Fiaz M., Farid M. A., Tahir M., Asghar M. A., Ul Hassan S., Hasan M. (2021). Iron and Manganese Codoped Cobalt Tungstates Co1-(x+y)Fe x Mn y WO4 as Efficient Photoelectrocatalysts for Oxygen Evolution Reaction. ACS Omega.

[cit27] Shivakumara C., Saraf R., Behera S., Dhananjaya N., Nagabhushana H. (2015). Scheelite-type MWO4 (M=Ca, Sr, and Ba) nanophosphors: Facile synthesis, structural characterization, photoluminescence, and photocatalytic properties. Mater. Res. Bull..

[cit28] Zin Elabedine G., Solé R. M., Slimi S., Aguiló M., Díaz F., Chen W., Petrov V., Mateos X. (2025). Growth, anisotropy, and spectroscopy of Tm3+ and Yb3+ doped MgWO4 crystals. CrystEngComm.

[cit29] Yang J., Xie T., Liu C., Xu L. (2018). Facile Fabrication of Dumbbell-Like β-Bi_2_O_3_/Graphene Nanocomposites and Their Highly Efficient Photocatalytic Activity. Materials.

[cit30] Kumar A., Sharma P., Wang T., Lai C. W., Sharma G., Dhiman P. (2024). Recent progresses in improving the photocatalytic potential of Bi4Ti3O12 as emerging material for environmental and energy applications. J. Ind. Eng. Chem..

[cit31] Kovalevskiy N., Cherepanova S., Gerasimov E., Lyulyukin M., Solovyeva M., Prosvirin I., Kozlov D., Selishchev D. (2022). Enhanced Photocatalytic Activity and Stability of Bi2WO6 - TiO2-N Nanocomposites in the Oxidation of Volatile Pollutants. Nanomaterials.

[cit32] Chen P., Liu H., Cui W., Lee S. C., Wang L. a., Dong F. (2020). Bi-based photocatalysts forlight-driven environmental and energy applications: Structural tuning, reaction mechanisms, and challenges. EcoMat.

[cit33] Nguyen H. C., Le P. D., Cao T. M., Pham V. V. (2024). Establishing Z-scheme Bi2WO6/g-C3N4 interfaces toward efficient photocatalytic performance of NOx under visible light. J. Alloys Compd..

[cit34] Shkir M., AlAbdulaal T. H., Ubaidullah M., Reddy Minnam Reddy V. (2023). Novel Bi2WO6/MWCNT nanohybrids synthesis for high-performance photocatalytic activity of ciprofloxacin degradation under simulated sunlight irradiation. Chemosphere.

[cit35] Nguyen P. H., Nguyen T. Q., Vo T. T. N., Cao T. M., Van Pham V. (2025). Advanced oxidation processes over Fe doped Bi2WO6 photocatalysts toward rhodamine B and cefalexin treatment. J. Sci.:Adv. Mater. Devices.

[cit36] Wang H., Liu H., Feng L., Yang D., Zhang C., Guo H., Wang Y., Wang H. (2025). Preparation and degradation performance of ag/bi2wo6/cofe2o4 ternary photocatalyst based on magnetic recovery. Langmuir.

[cit37] Feng L., Chen E., Li X., Wang J., Fan Y., Jin L., Tang P., Zhang L. (2025). High-performance 0D-2D S-scheme CdS-Bi2WO6 heterostructure for bifunctional photoelectrochemical detection and degradation of chlorpyrifos under visible light irradiation. Sep. Purif. Technol..

[cit38] Dhanaraman E., Verma A., Chen P. H., Chen N. D., Siddiqui Y., Fu Y. P. (2024). Bi2 WO6 Incorporation of g-C3 N4 to Enhance the Photocatalytic N2 Reduction Reaction and Antibiotic Pollutants Removal. Sol. RRL.

[cit39] Liu D., Chen M., Han Y., Sun C., Xu L., Su D. (2024). Enhanced directional charge transfer by 2D MXene/Bi2WO6/GO in visible light photocatalysis coupled persulfate approach for organic pollutants degradation. Sep. Purif. Technol..

[cit40] Morita K., Park J.-S., Kim S., Yasuoka K., Walsh A. (2019). Crystal Engineering of Bi2 WO6 to Polar Aurivillius-Phase Oxyhalides. J. Phys. Chem. C.

[cit41] Huang X., Soomro R. A., Shen H., Guo L., Yang C., Wang D. (2025). Bi2MO6 (M=Mo, W) Aurivillius Oxides for Efficient Photocatalytic N2-to-NH3 Conversion: A perspective review. Inorg. Chem. Front..

[cit42] Minohara M., Dobashi Y., Kikuchi N., Suzuki S., Samizo A., Honda T., Nishio K., Aiura Y. (2023). Control of hole density in russellite bi2wo6 *via* intentional chemical doping. Inorg. Chem..

[cit43] Bera S., Samajdar S., Pal S., Das P. S., Jones L. A. H., Finch H., Dhanak V. R., Ghosh S. (2022). Effect of metal doping in Bi2WO6 micro-flowers for enhanced photoelectrochemical water splitting. Ceram. Int..

[cit44] Zhang L.-Y., Yang J.-J., Han Y.-L. (2022). Novel adsorption-photocatalysis integrated bismuth tungstate modified layered mesoporous titanium dioxide (Bi2WO6/LM-TiO2) composites. Opt. Mater..

[cit45] Wang Y.-F., Xin F.-F., Deng Y.-R., Li D.-J., Li X.-F. (2020). Nano-Zn2SnO4/Reduced Graphene Oxide Composites for enhanced photocatalytic performance. Mater. Chem. Phys..

[cit46] Kospa D. A., Ahmed A. I., Samra S. E., Ibrahim A. A. (2021). High efficiency solar desalination and dye retention of plasmonic/reduced graphene oxide based copper oxide nanocomposites. RSC Adv..

[cit47] Gamal S., Kospa D. A., Kaid M. M., El-Hakam S. A., Ahmed A. I., Ibrahim A. A. (2023). Fe-Co spinel oxides supported UiO-66-NH2 derived zirconia/N-dopped porous hollow carbon as an efficient oxygen reduction reaction electrocatalyst. J. Environ. Chem. Eng..

[cit48] Ghanem R. M., Kospa D. A., Ahmed A. I., Ibrahim A. A., Gebreil A. (2023). Construction of thickness-controllable bimetallic sulfides/reduced graphene oxide as a binder-free positive electrode for hybrid supercapacitors. RSC Adv..

[cit49] Batool F., Muhammad S., Muazzam R., Waqas M., Ullah Z., Roy S., Wang K., Guo B. (2025). Advancements in Graphene-Based Composites: A Review of the Emerging Applications in Healthcare. Smart Mater. Med..

[cit50] Ha N. T. T., Ngo H. L., Pham T. B., Hoang Hao N., Bui C. T., Phung T. L., Cam L. M., Ngoc Ha N. (2024). Comprehensive Study on the Adsorption and Degradation of Dichlorodiphenyltrichloroethane on Bifunctional Adsorption-Photocatalysis Material TiO2/MCM-41 Using Quantum Chemical Methods. ACS Omega.

[cit51] Mitra M., Ahamed S. T., Ghosh A., Mondal A., Kargupta K., Ganguly S., Banerjee D. (2019). Polyaniline/Reduced Graphene Oxide Composite-Enhanced Visible-Light-Driven Photocatalytic Activity for the Degradation of Organic Dyes. ACS Omega.

[cit52] El-Yazeed W. S. A., El-Hakam S. A., Salama R. S., Ibrahim A. A., Ahmed A. I. (2022). Ag-PMA supported on MCM-41: Surface Acidity and Catalytic Activity. J. Sol. Gel Sci. Technol..

[cit53] Elias M., Alam R., Khatun S., Swapon Hossain M., Shaheen Shah S., Abdul Aziz M., Nizam Uddin M., Awlad Hossain M. (2025). Hydrothermal synthesis of carboxylated functionalized jute stick carbon and reduced graphene oxide based ZnO nanocomposite photocatalysts: A comparative study. J. Ind. Eng. Chem..

[cit54] Alomayri T. (2024). Enhanced interfacial charge transfer in BiVO4/rGO/FeVO4 heterojunction composite for improved photocatalysis water purification. Ceram. Int..

[cit55] Shyagathur S. C., Pattar J., Rao A. H. N., Sreekanth R., Mahendra K., Nagaraju G. (2024). Enhanced degradation of dyes using a novel CuS/g-C3N4/rGO ternary composite catalyst: Synthesis, characterization, and mechanistic insights. Mater. Chem. Phys..

[cit56] Prabakaran S., Nisha K. D., Harish S., Archana J., Navaneethan M. (2021). Yttrium incorporated TiO2/rGO nanocomposites as an efficient charge transfer layer with enhanced mobility and electrical conductivity. J. Alloys Compd..

[cit57] Singh D., Batoo K. M., Hussain S., Kumar A., Aziz Q. H., Sheri F. S., Tariq H., Singh P. (2024). Enhancement of the photocatalytic activity of rGO/NiO/Ag nanocomposite for degradation of methylene blue dye. RSC Adv..

[cit58] Alkhouzaam A., Qiblawey H., Khraisheh M., Atieh M., Al-Ghouti M. (2020). Synthesis of graphene oxides particle of high oxidation degree using a modified Hummers method. Ceram. Int..

[cit59] Manikandan M., Prasankumar T., Manikandan E., Papanasam E., Ramesh K., Ramesh S. (2024). Hydrothermal synthesis of rGO and MnCoS composite for enhanced supercapacitor application. Sci. Rep..

[cit60] Xu J., Ao Y., Chen M. (2013). A simple method for the preparation of Bi2WO6-reduced graphene oxide with enhanced photocatalytic activity under visible light irradiation. Mater. Lett..

[cit61] Huang C., Chen L., Li H., Mu Y., Yang Z. (2019). Synthesis and application of Bi2WO6 for the photocatalytic degradation of two typical fluoroquinolones under visible light irradiation. RSC Adv..

[cit62] Sharma S., Ibhadon A. O., Francesconi M. G., Mehta S. K., Elumalai S., Kansal S. K., Umar A., Baskoutas S. (2020). Bi2WO6/C-Dots/TiO2: A Novel Z-Scheme Photocatalyst for the Degradation of Fluoroquinolone Levofloxacin from Aqueous Medium. Nanomaterials.

[cit63] Balasurya S., Okla M. K., Al-amri S. S., Alaraidh I. A., Al-ghamdi A. A., Soufan W., Abdel-Maksoud M. A., Abdelaziz R. F., Studenik C. R., Khan S. S. (2022). Subsurface and solid solution-type defect engineering in the CoCr2 O4 –Bi2 WO4 –NiS2 nanocomposite for the visible-light degradation of doxycycline and removal of chromium and its genotoxic evaluation in Allium cepa. New J. Chem..

[cit64] Cao R., Huang H., Tian N., Zhang Y., Guo Y., Zhang T. (2015). Novel Y doped Bi2WO6 photocatalyst: Hydrothermal fabrication, characterization and enhanced visible-light-driven photocatalytic activity for Rhodamine B degradation and photocurrent generation. Mater. Charact..

[cit65] Dong H., Yin Y., Guo X. (2018). Synthesis and characterization of Ag/Bi2WO6/GO composite for the fast degradation of tylosin under visible light. Environ. Sci. Pollut. Res. Int..

[cit66] Ibrahim A. A., Ali S. L., Adly M. S., El-Hakam S. A., Samra S. E., Ahmed A. I. (2021). Green construction of eco-friendly phosphotungstic acid Sr-MOF catalysts for crystal violet removal and synthesis of coumarin and xanthene compounds. RSC Adv..

[cit67] Hu J., Ma J., Wang L., Huang H. (2014). Synthesis and photocatalytic properties of LaMnO3–graphene nanocomposites. J. Alloys Compd..

[cit68] Jiao J., Pan M., Liu X., Li B., Liu J., Chen Q. (2019). A Non-Enzymatic Sensor Based on Trimetallic Nanoalloy with Poly (Diallyldimethylammonium Chloride)-Capped Reduced Graphene Oxide for Dynamic Monitoring Hydrogen Peroxide Production by Cancerous Cells. Sensors.

[cit69] Wang R. T., Ma C. Y., Li J. R., Zhao G. H., Liu P. H., Qin F. W., Zhou N., Zhang Q. Y. (2024). Influence of substrate temperature on the structural, morphological, and photoelectric properties of Bi2WO6 thin films deposited by magnetron sputtering. Vacuum.

[cit70] Lin Z., Yang Z., Huang J. (2022). Hierarchical Bi2WO6/TiO2-nanotube composites derived from natural cellulose for visible-light photocatalytic treatment of pollutants. Beilstein J. Nanotechnol..

[cit71] Gamal S., Kospa D. A., Kaid M. M., El-Hakam S. A., Ahmed A. I., Ibrahim A. A. (2023). Fe-Co spinel oxides supported UiO-66-NH2derived zirconia/N-dopped porous hollow carbon as an efficient oxygen reduction reaction electrocatalyst. J. Environ. Chem. Eng..

[cit72] Tarek R., Kospa D. A., El-Hakam S. A., Ahmed A. I., Ibrahim A. A. (2023). Tailoring surface topography of biochar-based hydrogel for hazardous pollutants removal from contaminated seawater through simultaneous steam-electricity generation. Desalination.

[cit73] Basuny B. N., Kospa D. A., Ibrahim A. A., Gebreil A. (2023). Stable polyethylene glycol/biochar composite as a cost-effective photothermal absorber for 24 hours of steam and electricity cogeneration. RSC Adv..

[cit74] Li D., Yan P., Zhao Q., Bai X., Ma X., Xue J., Zhang Y., Liu M. (2020). Synthesis of bi2wo6/bi2moo6 heterostructured nanosheet and activating peroxymonosulfate to enhance photocatalytic activity. J. Inorg. Organomet. Polym..

[cit75] Li Y., Yu X., Li R., Zhao F., Liu G., Wang X. (2021). Selective and sensitive visible-light-prompt photoelectrochemical sensor of paracetamol based on Bi2WO6 modified with Bi and copper sulfide. RSC Adv..

[cit76] Li H., Zhang J., Yu J., Cao S. (2021). Ultra-Thin Carbon-Doped Bi2WO6 Nanosheets for Enhanced Photocatalytic CO2 Reduction. Trans. Tianjin Univ..

[cit77] Shafaati M., Miralinaghi M., Shirazi R. H. S. M., Moniri E. (2020). The use of chitosan/Fe3O4 grafted graphene oxide for effective adsorption of rifampicin from water samples. Res. Chem. Intermed..

[cit78] Aragaw T. A., Alene A. N. (2022). A comparative study of acidic, basic, and reactive dyes adsorption from aqueous solution onto kaolin adsorbent: Effect of operating parameters, isotherms, kinetics, and thermodynamics. Emerging Contam..

[cit79] da Silva L. F., Lopes O. F., de Mendonça V. R., Carvalho K. T. G., Longo E., Ribeiro C., Mastelaro V. R. (2016). An Understanding of the Photocatalytic Properties and Pollutant Degradation Mechanism of SrTiO3 Nanoparticles. Photochem. Photobiol..

[cit80] Noor T., Yaqoob L., Iqbal N. (2021). Recent Advances in Electrocatalysis of Oxygen Evolution Reaction using Noble-Metal, Transition-Metal, and Carbon-Based Materials. ChemElectroChem.

[cit81] Liu X., Zhang Q., Li J., Valanoor N., Tang X., Cao G. (2018). Increase of power conversion efficiency in dye-sensitized solar cells through ferroelectric substrate induced charge transport enhancement. Sci. Rep..

[cit82] Wu W., Yan Z., Wang L., Zhu X., Zhu Y., Liao G., Zhu L. (2024). Efficient wo3 nanoplate arrays photoanode modified by zno nanosheets for enhanced charge separation and transfer to promote photoelectrochemical performances. Adv. Electron. Mater..

[cit83] Zargazi M., Entezari M. H. (2020). Ultrasound assisted deposition of highly stable self-assembled Bi2MoO6 nanoplates with selective crystal facet engineering as photoanode. Ultrason. Sonochem..

