# Supplementary material for: Reduced graphene oxide/ bismuth tungstate-based photocatalysts for enhanced dye photodegradation and photoelectrochemical water splitting
Source: RSC Adv. 2025 Jul 25;15(33):26608–22. doi: 10.1039/d5ra04049c (PMC12292039; doi:10.1039/d5ra04049c)
Supplement: RA-015-D5RA04049C-s001 [file RA-015-D5RA04049C-s001.pdf]

Supplementary Information (SI) for RSC Advances.
This journal is © The Royal Society of Chemistry 2025

# Reduced graphene oxide/ Bismuth Tungstate-based photocatalysts for enhanced dye photodegradation and photoelectrochemical water splitting

**Amr Awad Ibrahim[a*], Doaa A. Kospa[a*], Salah Orabi[a], Salma M. Abo Kamar[a], Ahmed A. Salah[a], E. A. El-Sharkawy[b], S. A. El-Hakam[a], Awad I. Ahmed[a]**

[a]*Department of Chemistry, Faculty of Science, Mansoura University, Al-Mansoura 35516, Egypt.*

[b]*Department of Chemistry, Faculty of Science, Suez University, Suez, Egypt*

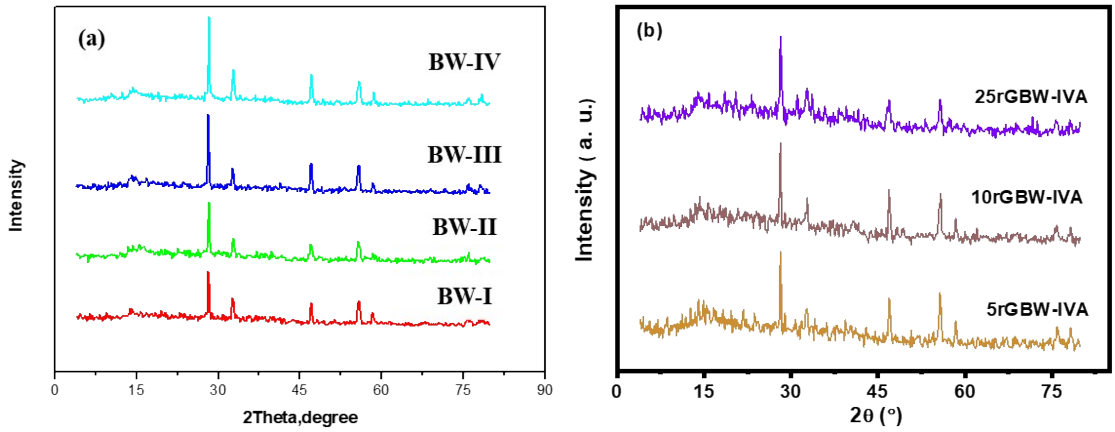


**Fig. S1:** XRD patterns of (a) $Bi_2WO_6$ calcined at different temperatures and (b) the $Bi_2WO_6$ composites with different ratios of rGO.

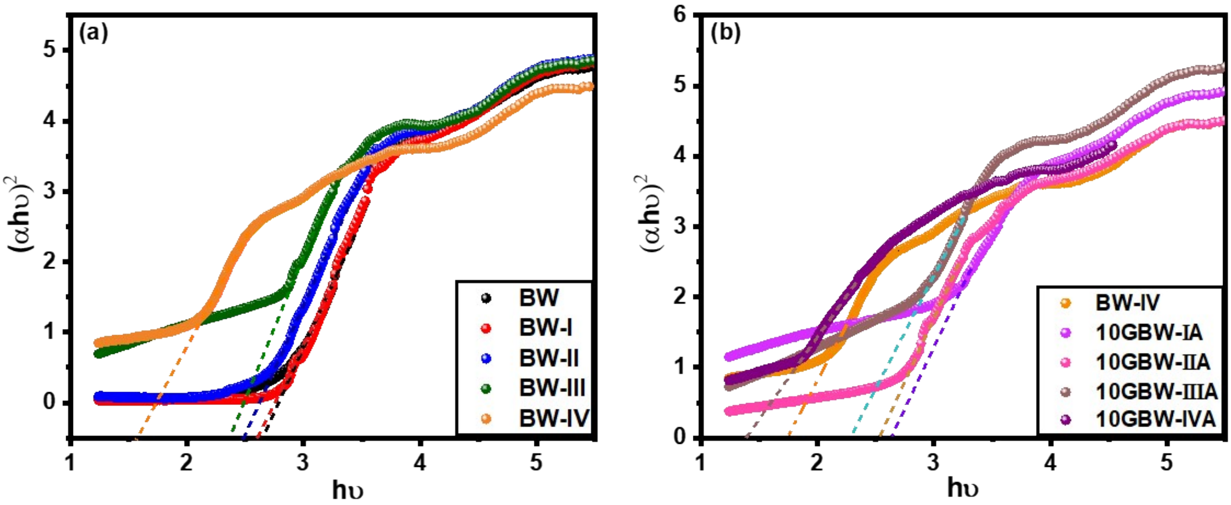


**Fig. S2:** Plot of $(\alpha h\nu)^2$ versus (hν) for (a) $Bi_2WO_4$ and (b) rGO/$Bi_2WO_4$ composites.

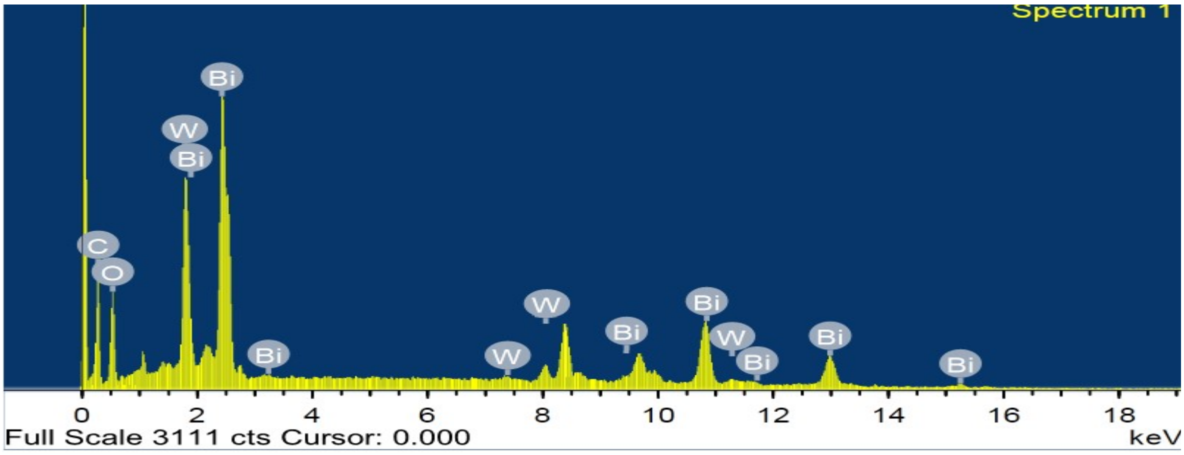

**Fig. S3:** The energy dispersive X-ray spectroscopy (EDX) analysis of 10rGBW-IVA.

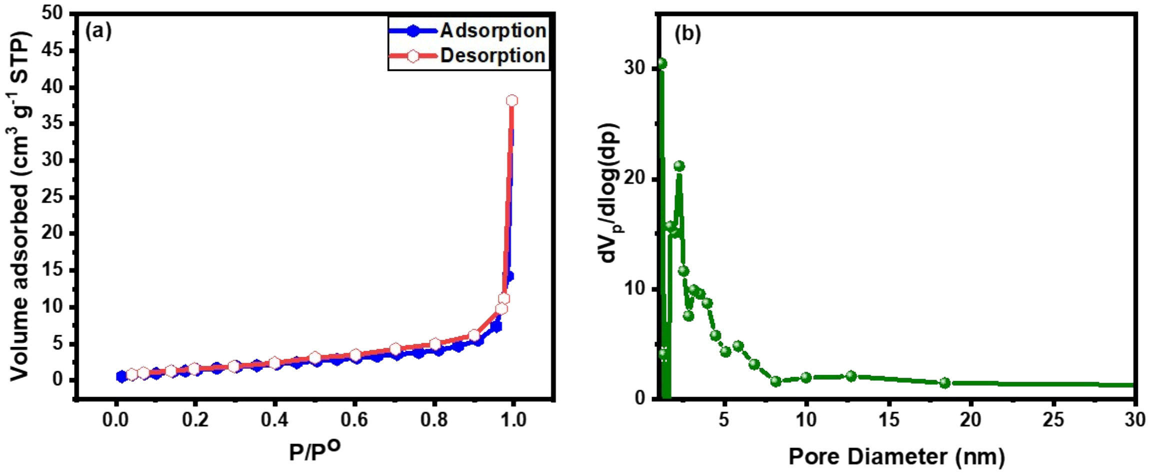


**Fig. S4:** (a) Nitrogen adsorption–desorption isotherm of the 10rGBW-IVA at 77 K. (b) Pore size distribution curve derived from the adsorption branch using the BJH (Barrett–Joyner–Halenda) method.

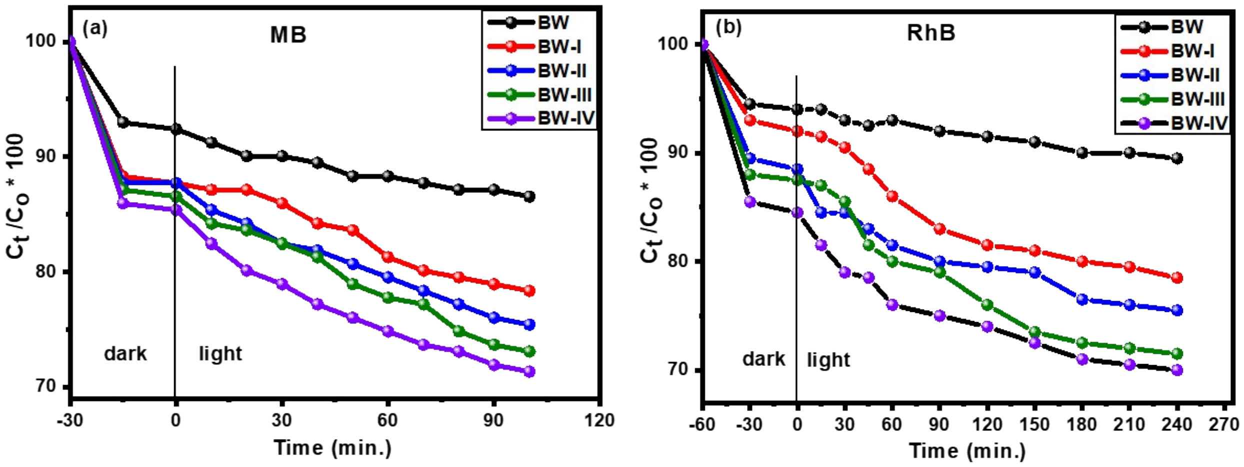

**Fig. S5:** %Degradation of (a) MB and (b) RhB using bithmus tungestate at different calcination temperatures after 100 min of light irradaiation.

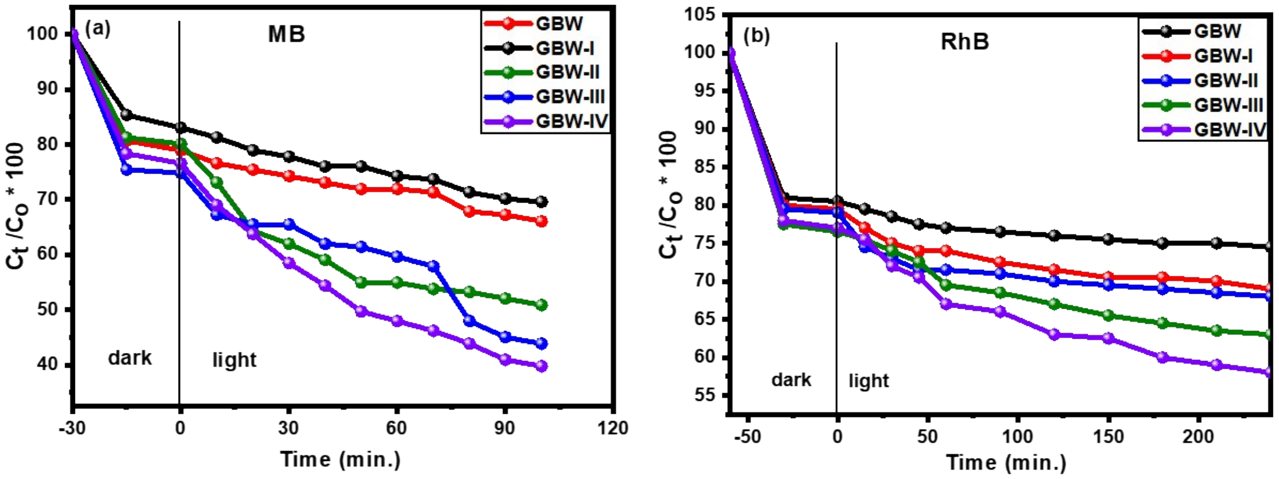


**Fig. S6:** %Degradation of (a) MB and (b) RhB using graphene doped bithmus tungestate at different temperatures after 100 min of light irradaiation.

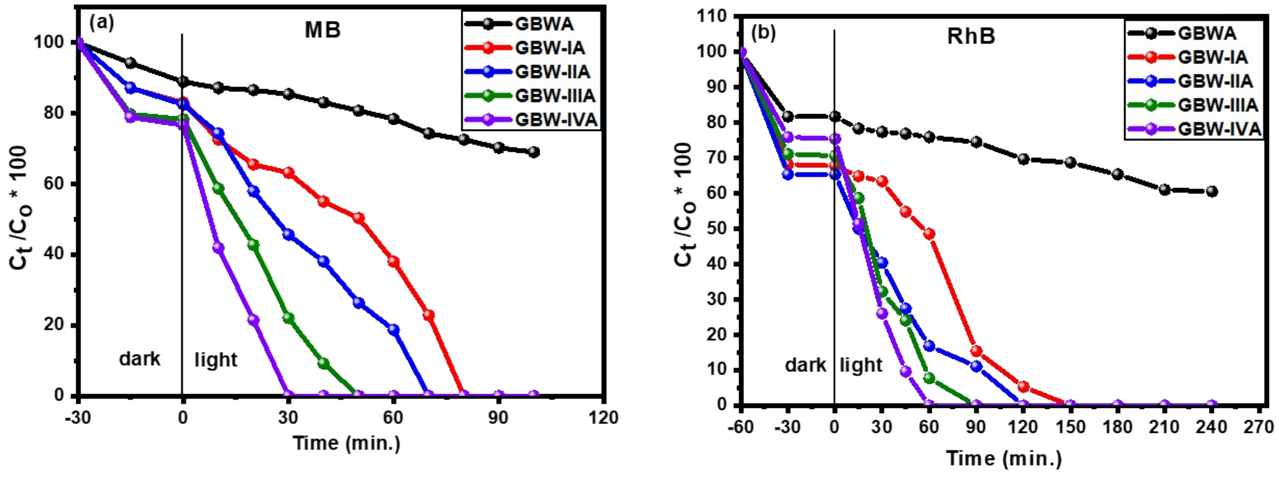


**Fig. S7:** %Degradation of (a) MB and (b) RhB using GBW-X at calcination temperature of 250 °C after 100 min of light irradaiation.

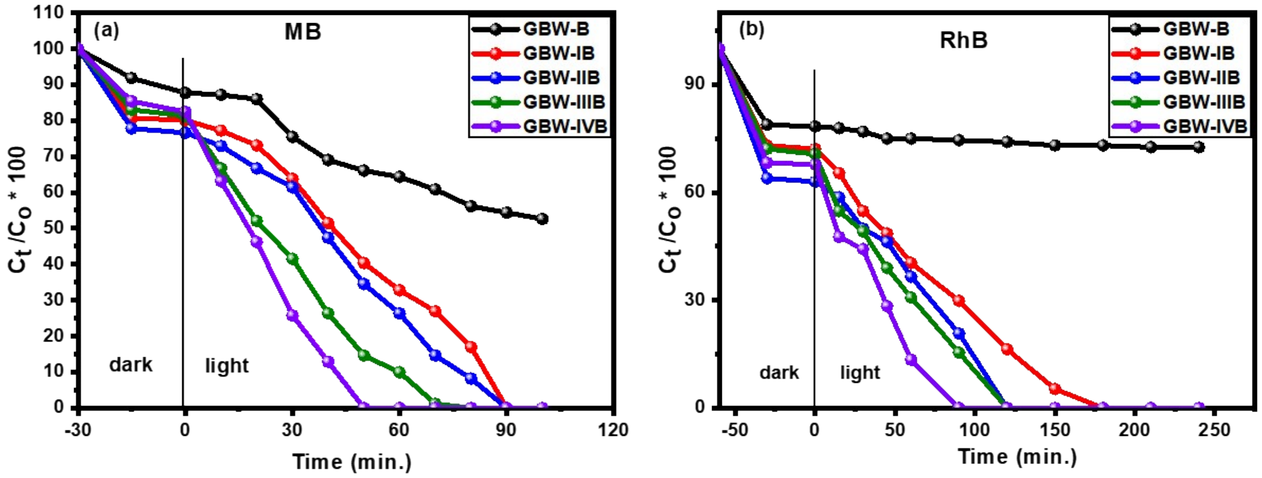


**Fig. S8:** %Degradation of (a) MB and (b) RhB using GBW-X at calcination temperature of 350 °C after 100 min of light irradaiation.

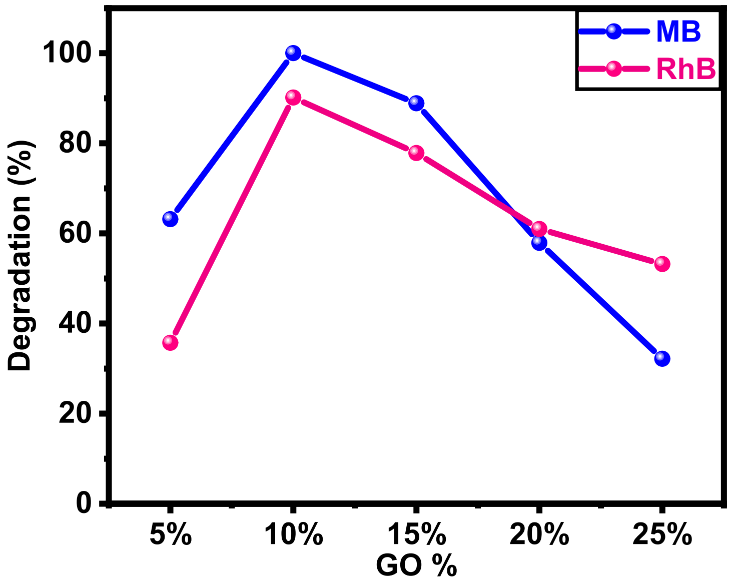


**Fig. S9:** %Degradation of MB and RhB using different amounts of GO.

**Table. S1:** The %photocatalytic degradation of MB and RhB by different photocatalysts after 40 min.

| Sample | % degradation | | Sample | % degradation | | Sample | % degradation | | Sample | % degradation | |
|---|---|---|---|---|---|---|---|---|---|---|---|
| | **MB** | **RhB** | | **MB** | **RhB** | | **MB** | **RhB** | | **MB** | **RhB** |
| BW-I | 15.8 | 11.5 | 10rGBW-I | 23.9 | 25.9 | 10rGBW-IA | 54.9 | 45.2 | 10rGBW-IB | 42.5 | 39.4 |
| BW-II | 18.1 | 16.2 | 10rGBW-II | 40.9 | 28.5 | 10rGBW-IIA | 71.9 | 62.6 | 10rGBW-IIB | 52.6 | 44.8 |
| BW-III | 19.7 | 17.5 | 10rGBW-III | 48.1 | 37.5 | 10rGBW-IIIA | 90.8 | 75.9 | 10rGBW-IIIB | 73.7 | 61.1 |
| BW-IV | 22.8 | 21.5 | 10rGBW-IV | 55.6 | 45.1 | 10rGBW-IVA | 100.0 | 87.6 | 10rGBW-IVB | 88.1 | 71.6 |
| 5rGBW-IVA | 73.7 | 64.8 | 15rGBW-IVA | 80.4 | 69.6 | 20rGBW-IVA | 66.8 | 54.3 | 25rGBW-IVA | 45.7 | 34.9 |

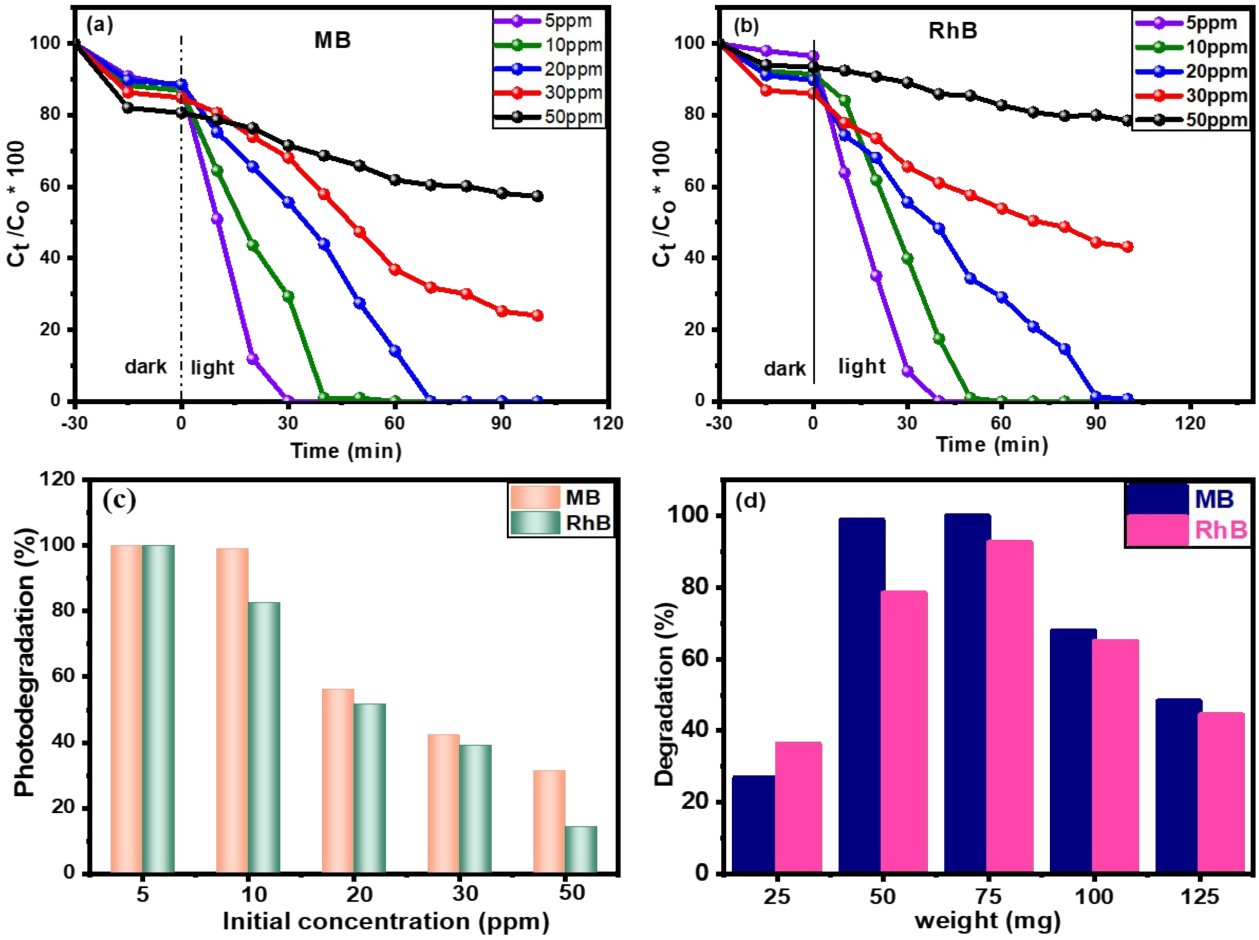


**Fig. S10: Photodegradation** of (a) MB and (b) RhB and (c) % degradation of dyes using 75 mg of 10GBWIV-A with different concentrations of dyes after 100 min of light irradiation, and (d) % degradation of dyes using different doses of 10GBWIV-A with 100 ppm of dyes after 100 min of light irradiation.

**Table S2:** First order $k_{app}$ values for photocatalytic degradation of MB and RhB.

| rGBW-IVA | MB | | RhB | |
|---|---|---|---|---|
| | $K_{app}$ ($min^{-1}$) | $R^2$ | $K_{app}$ ($min^{-1}$) | $R^2$ |
| 5rGBW-IVA | 0.008 | 0.98455 | 0.007 | 0.98747 |
| 10rGBW-IVA | 0.039 | 0.99963 | 0.030 | 0.99894 |
| 15rGBW-IVA | 0.025 | 0.99975 | 0.021 | 0.99987 |
| 20rGBW-IVA | 0.018 | 0.9977 | 0.015 | 0.99623 |
| 25rGBW-IVA | 0.015 | 0.99856 | 0.012 | 0.99494 |

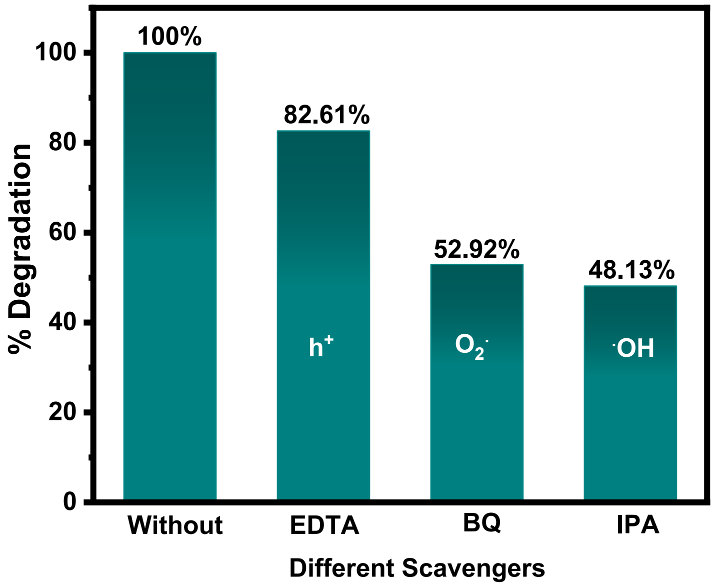


**Fig. S11:** The influence of various radical scavengers on the photocatalytic degradation efficiency of the catalyst.

**Table. S3:** A comparison of photocurrent density values for $Bi_2WO_6$-based and graphene-based photocatalytic materials reported in the literature.

| Materials | Photocurrent density (μA/cm$^2$) | Ref. |
|---|---|---|
| **$LaCoO_3/Bi_2WO_6$** | ~0.1 | 1 |
| **$Cu/Bi_2WO_6$** | 0.95 | 2 |
| **$Ag_2MoO_4/Bi_2WO_6$** | 0.5 | 3 |
| **$Bi_2WO_6$/MIL** | ~0.1 | 4 |
| **$Bi_2WO_6$/Na-bentonite** | ~0.1 | 5 |
| **$BiVO_4$/RGO** | 1.3 | 6 |
| **$TiO_2$/Graphene** | ~1.0 | 7 |
| **10rGBW-IVA** | **~1.4** | **This work** |

## References


1. Guo, J.; Xu, Y.; Li, J.; Yang, C.; Lu, C., Preparation of a novel composite material LaCoO3/Bi2WO6 and its application in the treatment of tetracycline. *J Mater Sci: Mater Electron* **2021,** *32* (10), 13813-13824.
2. Li, J.; Liang, Z.; Qin, Y.; Guo, L.; Lei, N.; Song, Q., Defective bi2 wo6 -supported cu nanoparticles as efficient and stable photoelectrocatalytic for water splitting in near-neutral media. *Energy Technology* **2018,** *6* (11), 2247-2255.
3. Gu, Y.; Guo, B., Preparation and study of Z-type heterojunction composite photocatalytic material with Ag2MoO4-modified rosette-like Bi2WO6 with Ag-SPR-promoting effect. *J Mater Sci: Mater Electron* **2021,** *32* (10), 13305-13322.
4. Hu, L.; Zhang, Y.; Lu, W.; Lu, Y.; Hu, H., Easily recyclable photocatalyst Bi2WO6/MOF/PVDF composite film for efficient degradation of aqueous refractory organic pollutants under visible-light irradiation. *J Mater Sci* **2019,** *54* (8), 6238-6257.
5. Yang, Q.; Dai, Y.; Huang, Z.; Zhang, J.; Zeng, M.; Shi, C., Synthesis of Bi2WO6/Na-bentonite composites for photocatalytic oxidation of arsenic(iii) under simulated sunlight. *RSC Adv* **2019,** *9* (51), 29689-29698.
6. Sun, J.; Wang, C.; Shen, T.; Song, H.; Li, D.; Zhao, R.; Wang, X., Engineering the Dimensional Interface of BiVO4-2D Reduced Graphene Oxide (RGO) Nanocomposite for Enhanced Visible Light Photocatalytic Performance. *Nanomaterials (Basel)* **2019,** *9* (6).
7. Ye, H.; Wang, Z.; Liu, Y.; Chen, S.; Wang, H.; Wu, Z., Efficient Degradation of Gas-Phase Toluene by Ozone-Assisted Photocatalytic Oxidation on TiO2/Graphene Composites. *Catal. Lett* **2019,** *149* (10), 2739-2748.
